# Melissospora conviva gen. nov., sp. nov., a novel actinobacterial genus isolated from beehive through cross-feeding interactions

**DOI:** 10.1099/ijsem.0.006868

**Published:** 2025-08-11

**Authors:** Déborah Tellatin, Luc Cornet, Valdes Snauwaert, Philippe Compère, Marc Ongena, Loïc Quinton, Nudzejma Stulanovic, Silvia Ribeiro Monteiro, Augustin Rigolet, Pierre Burguet, Petra Van Damme, Lorena Carro, Sébastien Rigali

**Affiliations:** 1 CIP, InBioS – Center for Protein Engineering, University of Liège, Institut de Chimie, Liège B-4000, Belgium; 2 BCCM/ULC, InBioS – Molecular Diversity and Ecology of Cyanobacteria, University of Liège, Liège B-4000, Belgium; 3 iR.I.P. Unit – Laboratory of Microbiology, University of Ghent, Ghent B-9000, Belgium; 4 Laboratory of Functional and Evolutionary Morphology, University of Liège, Liège B-4000, Belgium; 5 Microbial Processes and Interactions Laboratory, TERRA Teaching and Research Centre, Gembloux Agro-Bio Tech, University of Liège, Gembloux B-5030, Belgium; 6 Mass Spectrometry Laboratory, MolSys Research Unit, University of Liège, Liège-B 4000, Belgium; 7 Department of Molecular Biotechnology, Institute of Biology, Leiden University, Leiden 2311 EZ, Netherlands; 8 Departamento de Microbiología y Genética, Universidad de Salamanca, Salamanca 37008, Spain

**Keywords:** auxotrophy, bioprospecting, insect-associated bacteria, microbial cross-feeding, microbial dark matter, uncultured bacteria

## Abstract

Most micro-organisms remain unculturable under standard laboratory conditions, limiting our understanding of microbial diversity and ecological interactions. One major cause of this uncultivability is the loss of access to essential cross-fed metabolites when bacteria are removed from their natural communities. During a bioprospecting campaign targeting actinomycetes of an *Apis mellifera* beehive, we identified five isolates (DT32, DT45^T^, DT55, DT59 and DT194) that required co-cultivation for growth recovery, suggesting a dependence on microbial interactions in their native habitat. Whole-genome sequencing and phylogenetic analysis positioned these isolates within a distinct lineage of *Micromonosporaceae*, separate from the five officially recognized clades of the *Micromonospora* genus. A combination of microscopic, chemotaxonomic and physiological characterizations further supported their uniqueness. Notably, they exhibited high auxotrophy, being unable to use all carbon sources tested, likely due to genome reduction (4.6 Mbp) compared to other *Micromonosporaceae*. Pangenomic comparisons with their closest *Micromonospora* relatives revealed gene losses in key metabolic pathways, including the glyoxylate bypass and the Entner–Doudoroff pathway, which may explain their metabolic reliance. These findings reveal a highly specialized, ecologically adapted lineage with deep evolutionary divergence and further support microbial interdependence isolation strategies to explore the microbial dark matter. We propose *Melissospora conviva* as a novel genus and species within the *Actinomycetota* phylum, with isolate DT45^T^ as the representative type species and type strain, which has been deposited in public collections under the accession numbers DSM 117791 and LMG 33580.

## Introduction

The bioprospecting of micro-organisms has become an essential strategy for gaining deeper insight into microbial diversity and for uncovering novel bioactive compounds. Micro-organisms inhabit virtually every environment on Earth, including extreme and previously considered uninhabitable niches, where they exhibit unique physiological and metabolic adaptations. A significant portion of this microbial diversity – often referred to as microbial dark matter – remains difficult to culture by conventional methods, limiting our understanding of their biology and ecological roles. Accessing and characterizing these elusive organisms is not only crucial for identifying new functional biomolecules but also for advancing our knowledge of microbial physiology, evolution and ecosystem functioning [1,2].

During our previous bioprospecting campaigns aimed at isolating actinobacteria adapted to cave moonmilk deposits, we frequently encountered the loss of certain isolates during the subculturing process, in a proportion that could reach nearly 20% of the colonies transplanted [3,4]. This loss occurred when bacteria transferred from the initial environmental isolation dishes failed to grow as axenic cultures. Losing bacterial isolates during subculturing is problematic because it reduces the diversity of the culture collection, potentially eliminating rare or unique strains with valuable physiological or biotechnological traits. This loss limits downstream analyses, such as genomic or metabolomic characterization, and can bias the interpretation of microbial diversity in the original environment. In this study, we present an example of such an instance, where co-cultivation of highly auxotrophic isolates allowed growth rescue. Comparative analyses revealed a high taxonomy level of endemism, suggesting a new genus within the *Actinomycetota* phylum, for which we propose the name *Melissospora conviva*.

## Methods

### Strain isolation and culture conditions

Honeybee (*Apis mellifera*) products, including pollen, wax, propolis, honey and the surface of a bee, were collected aseptically and separately in falcon tubes from a hive located in Comblain-au-Pont, Belgium (50° 29′ 19.6″ N 5° 33′ 45.1″ E), in May 2021. The samples were soaked in PBS (0.01 M, pH 7.0), and serial dilutions were inoculated onto selective isolation medium supplemented with nalidixic acid (75 µg ml^−1^) and nystatin (50 µg ml^−1^) to inhibit the growth of Gram-negative bacteria and fungi, respectively. The isolation medium used was the International *Streptomyces* Project (ISP) medium 5 (ISP5 [5]), with phosphate and agar autoclaved separately (ISP5-S) to prevent reactive oxygen species production [3]. After two rounds of subculture, the biomass of isolates was amplified in Müller–Hinton broth 2 (MHB, Millipore^®^) liquid cultures at 28 °C and stored in glycerol mycelium stocks (20%, w/v) at −20 and −80 °C. The strains used in this study are listed in Table 1. For growth monitoring and phenotype characterization, 100 µl of a 1-week MHB preculture was streaked and incubated at 28 °C for 1 week on solid Müller–Hinton agar (MHA, Millipore^®^), tryptic soy agar (TSA, Millipore^®^), Luria–Bertani (LB, VWR Chemicals), ISP media 1, 2, 5, 6 and 7 [5], Yeast-Malt (YM [6]) and R2YE [7] media.

**Table 1.** Strains used in this study

Name	Strain	Comment	Source	Genome/16S rRNA accession
*Melissospora conviva*	DT45^T^	Auxotrophic strains isolated on ISP5-S, type strain	Pollen	JBLLDX000000000/PV139158
*Melissospora conviva*	DT32	Auxotrophic strains isolated on ISP5-S	Bee	JBLLDW000000000/PV139159
*Melissospora conviva*	DT55		Pollen	JBLLDT000000000/PV139160
*Melissospora conviva*	DT59		Pollen	JBLLDU000000000/PV139204
*Melissospora conviva*	DT194		Propolis	JBLLDV000000000/PV139205
*Micromonospora* sp.	DT15	Autotrophic strain isolated on ISP5-S	Bee	JBNCKJ000000000/PV164011
*Micromonospora arida*	DT42	Isolate neighbouring DT45^T^ in the original ISP5-S plate	Pollen	JBNCKI000000000/PV164012
*Micromonospora* sp.	DT43		Pollen	JBNCKR000000000/PV164014
*Micromonospora* sp.	DT44		Pollen	JBNCKS000000000/PV164015
*Micromonospora arida*	DT56	Isolate neighbouring DT59 in the original ISP5-S plate	Pollen	JBNCOC000000000/PV164016
*Actinomadura citrea*	DT60	Isolate neighbouring DT32 in the original ISP5-S plate	Pollen	JBNCNN000000000/PV164017
*Peribacillus castrilensis*	DT69	Lab contamination that rescued the growth of DT45^T^	Unknown	JBNBLK000000000/PV164018
*Streptomyces* sp.	DT190	Isolate neighbouring DT194 in the original ISP5-S plate	Propolis	JBNCNM000000000/PV164024

### Scanning electron microscopy

Strain DT45^T^ was cultivated on MHA plates for a week at 28 °C. After cultivation, cells were fixed in 1% osmium tetroxide (OsO_4_) in distilled water and sequentially dehydrated through a graded ethanol series (30–100%). Both glutaraldehyde-fixed and ethanol-preserved samples were then processed by critical point drying. The dried samples were mounted on glass slides covered by double-sided carbon tape as performed previously [8]. Subsequently, the samples were sputter-coated with platinum using a Balzers sputtering unit SCD 030 (Balzers, Lichtenstein). Scanning electron micrographs of the platinum-coated samples were obtained using a TESCAN Clara S8124 under high vacuum conditions, with the Everhart-Thornley (ET)-secondary electron detector at a 10 mm working distance and 15 kV accelerating voltage.

### Cross-feeding experiments

Co-inoculation experiments were conducted by streaking strains DT32, DT45^T^, DT55, DT59 and DT194 with 5 µl of a mycelium stock onto ISP5-S medium. Additionally, 25 µl of the nearest neighbouring strains from the original isolation plate (DT42, DT43, DT44, DT56, DT60 and DT190) was spotted at the centre of the ISP5-S plate and incubated at 28 °C for 1 month. Neighbouring strains were also spotted in close proximity to spots of DT45^T^ strain, each 1 cm apart, on ISP5-S solid medium under the same incubation conditions (28 °C for 1 month).

To condition ISP5-S medium with neighbouring strains, 50 µl of a 1-week MHB preculture of strains DT42, DT43, DT44, DT56, DT60 and DT190 was streaked onto a cellophane disc (Gel Company, Inc.) and incubated at 28 °C for 1 week. After incubation, the cellophane containing the strain mycelia was removed, and 100 µl of strain DT45^T^ was inoculated across the entire plate. The plates were then incubated at 28 °C for 1 month. The culture supernatant of strain DT69 was obtained after 1 week of incubation on ISP5-S solid medium at 28 °C. The full extract was collected through a freeze-thaw cycle, and the supernatant was filtered using 0.22 µm filter discs.

### Chemotaxonomic characterization

The analysis of respiratory menaquinones, polyamines, whole-cell sugars, fatty acids, mycolic acids, polar lipids and peptidoglycan structure was carried out by DSMZ Services at the Leibniz-Institut DSMZ – Deutsche Sammlung von Mikroorganismen und Zellkulturen GmbH, Braunschweig, Germany. Respiratory menaquinones were identified using HPLC coupled with diode-array detection and MS (HPLC-DAD-MS). Polyamines were extracted from 50 to 60 mg of wet biomass according to Zech *et al*. [9] and analysed via GC-MS. The polyamines and precursors screened included agmatine, cadaverine, homospermidine, norspermidine, 1,2- and 1,3-diaminopropane, putrescine, *N*-acetyl-putrescine, 2-hydroxyputrescine, spermidine and spermine. The analysis of peptidoglycan structure followed the published protocols of Schumann [10]. Cellular fatty acids are analysed after conversion into fatty acid methyl esters (FAMEs) by saponification, methylation and extraction following the protocol of Sasser [11]. The FAME mixtures are separated by GC and detected by a flame ionization detector. In subsequent analysis, fatty acids are identified by a GC-MS run on an Agilent GC-MS 7000D system [12]. Mycolic acids are extracted from wet biomass using minor modifications of the method described by Vilchèze and Jacobs [13] for analysis of mycolic acids by HPLC. Dried extracts are reconstituted in chloroform:methanol and analysed on an Agilent QT of mass spectrometer by direct infusion into the Electrospray Ionization (ESI) source. Polar lipids are extracted from freeze-dried cell material using a chloroform:methanol:0.3% aqueous NaCl mixture; polar lipids are recovered into the chloroform phase (modified after Bligh and Dyer [14]). Polar lipids are separated by two-dimensional silica gel TLC. The first direction is developed in chloroform:methanol:water, and the second in chloroform:methanol:acetic acid:water. Total lipid material is detected using molybdatophosphoric acid, and specific functional groups are detected using spray reagents specific for defined functional groups [15].

### Phenotypic and biochemical characterization

The carbon source utilization pattern was determined by inoculating isolates DT45^T^ and DT15 from a 1-week preculture in MHB, which was then washed twice and suspended in sterile water. This suspension was then placed on minimal medium (MM [7]) supplemented with 1% d-glucose, d-ribose, d-fructose, d-xylose, d-galactose, d-raffinose, d-maltose, d-cellobiose, d-mannitol, d-melezitose, d-mannose, d-trehalose, l-sorbose, l-arabinose, l-rhamnose, *N*-acetylglucosamine, citric acid, malic acid or water as a negative control. Tolerance to a range of NaCl concentrations (1–7%, at 1% intervals), pH levels (5.0–10.0, at 1.0 pH unit intervals) and temperatures (4, 12, 20, 28, 37 and 41 °C) was quantified after 1 week of cultivation in MHB liquid medium.

### DNA sequencing

Each axenic isolate was cultivated in MHB liquid medium at 28 °C, and genomic DNA was extracted using the GenElute Bacterial Genomic DNA kit (Sigma-Aldrich, St. Louis, MO, USA), according to the manufacturer’s instructions. The quality and concentration of gDNA were analysed using a Nanodrop and by electrophoresis on 1% agarose gels at 100 V for 30 min. Genomic libraries were constructed using Illumina Tagmentation DNA PCR-free (Illumina, Inc., San Diego, CA, USA). Whole-genome sequencing was performed on the Illumina NovaSeq S4 v1.5 platform with a 300-cycle XP workflow (1 lane). Complete genomes were assembled *de novo* from raw sequence data with SPAdes v3.14.0 [16], using the ‘careful’ mode. In addition, the full-length sequence of the 16S rRNA gene of strain DT45^T^ was PCR amplified using primers rRNA 16S forward (16 S-27F – AGAGTTTGATCCTGGCTCAG) and rRNA 16S reverse (16 S-1541R – AAGGAGGTGATCCAGCCGCA) and Sanger-sequenced. Multiple sequence alignment using clustal Omega (clustalo 1.2.4) [17] showed that the PCR amplified a 1,034 bp long 16S rRNA gene sequence from strain DT45^T^, which was identical to the 16S rRNA gene sequences retrieved from genome sequencing.

### Phylogenomic analyses

The 16S rRNA gene sequences and whole genomes were compared against all available type strain genomes in the type (strain) genome server (TYGS) [18] database using the MASH algorithm, a rapid method for estimating intergenomic relatedness. For the genomes of strains DT32, DT45^T^, DT55, DT59 and DT194, the ten type strains with the smallest MASH [19] distances were selected. Additionally, a second set of ten closely related type strains was identified based on 16S rRNA gene sequences. These sequences were extracted from the genomes with RNAmmer [20] and subsequently aligned using blast against the 16S rDNA sequences of each of the 19,435 type strains in the TYGS database. This approach served as a proxy for identifying the 50 best-matching type strains (based on bitscore) for each genome. These selected strains were then used to compute precise distances through the genome blast distance phylogeny (GBDP) approach under the ‘coverage’ algorithm and distance formula d_5_. The ten closest type strain genomes for each of the DT32, DT45^T^, DT55, DT59 and DT194 strains were subsequently determined.

For phylogenomic inference, pairwise comparisons of the genomes of the DT32, DT45^T^, DT55, DT59 and DT194 strains were performed using GBDP, with accurate intergenomic distances calculated under the ‘trimming’ algorithm and distance formula d_5_. Each comparison included 100 distance replicates. Digital DNA–DNA hybridization (dDDH) values and confidence intervals were calculated using the recommended settings of the genome-to-genome distance calculator (GGDC 4.0). The resulting intergenomic distances were used to construct a balanced minimum evolution tree, with branch support calculated via FastME 2.1.6.1 [21], including Subtree Pruning and Regrafting (SPR) postprocessing. Branch support values were inferred from 100 pseudo-bootstrap replicates. The resulting trees were rooted at the midpoint and visualized using the Interactive Tree of Life (iTOL [22]).

The use of distance formula d_4_ was preferred, according to developers’ recommendation, as it is independent of genome length and is therefore robust when analysing genomes obtained via Illumina sequencing, which often results in incomplete draft genomes.

The complete set of *Actinomycetota* genomes, comprising 46,471 genomes, was downloaded using the genome-downloader workflow (version 3.0.0 from the GENERA suite [23], 22 July 2023). The genomes of the DT32, DT45^T^, DT55, DT59 and DT194 strains were then compared against this dataset using the average nucleotide identity (ANI) workflow (version 3.0.0) from GENERA, with fastANI (version 1.3.3) [24]. A total of 86 genomes with an ANI value of 81% or higher were retained for further analyses. To this dataset, all previously downloaded *Micromonosporales* genomes were added to create a comprehensive dataset for phylogenomic analyses. These genomes were analysed using the GENcontams workflow, version 3.0.0, from GENERA, with CheckM (version 1.2.2) [25] and GUNC (version 1.0.5) [26], following the complementary contamination detection recommendations of Cornet and Baurain [27]. Genomes with 5% or more contamination and less than 90% completeness were excluded, resulting in a dataset of 442 genomes. Additionally, 12 *Rubrobacterales* genomes, decontaminated using the same criteria, were included as an outgroup. This resulted in a dataset of 459 genomes (442 selected genomes, 12 outgroup genomes and the 5 DT strains). This dataset was processed using the Orthology workflow (version 2.0.8) from GENERA with the OrthoFinder [28] option (version 2.5.4) and the core genes setting activated, identifying 81 core genes. These 81 core genes were analysed using the Phylogeny workflow (version 2.0.0) from GENERA. Sequence alignments were performed with MAFFT (version 7.453) [29], ambiguous site selection was done with BMGE (version 1.12) [30] and concatenation and matrix generation were conducted using SCaFoS (version 1.30) [31]. This resulted in a matrix of 459 sequences spanning 8,461 positions, with 12.03% missing states. The phylogenomic tree, available upon request, was inferred using RAxML (version 8.1.17) [32] under the PROTGAMMALGF model via the Phylogeny workflow. A manually selected subset of 133 genomes was defined based on this tree, and a new orthology analysis, using the same parameters as described above, identified 260 core genes. A subsequent phylogenomic analysis, using the same workflow and parameters as described above, produced an alignment matrix of 133 sequences spanning 58,435 positions with 0.45% missing states. This resulting large phylogenomic tree, rooted on *Actinoplanes*, was inferred as described above. Finally, a focused dataset of 26 genomes, comprising the node corresponding to the DT strains and the outgroup, was used to identify 388 core genes. This analysis produced a matrix of 26 sequences over 73,253 positions with 3.11% of missing states, analysed as described above. ANI heatmaps were generated using ggplot2, based on the outputs from the ANI workflow. The ecological analyses of the strain DT45^T^ were performed using the Protologger web tool [33].

### Pangenomic analysis

A representative selection of 17 *Micromonospora* genomes was analysed using the pangenomic workflow of anvi’o, version 8 [34]. The following scripts were used: anvi-script-reformat-fasta (with the simplify-names option activated), anvi-gen-contigs-database, anvi-run-ncbi-cogs, anvi-gen-genomes-storage, anvi-pan-genome and anvi-display-pan. These scripts enabled the visualization of clusters of orthologous groups pathways for the selected genomes.

### Identification and analysis of biosynthetic gene clusters

The genome of strains DT32, DT45^T^, DT55, DT59 and DT194 was subjected to analysis by the Antibiotics and Secondary Metabolite Analysis Shell software (antiSMASH version 7.1.0 [35]) for biosynthetic gene clusters (BGCs) prediction. BGC’s sequence similarity networks were constructed and compared to the minimum information about a BGC (MIBiG version 3.0 [36]) repository using the Biosynthetic Gene Similarity Clustering and Prospecting Engine software (BiG-SCAPE version 1.1.5 [37]) with a threshold of 0.65. The network was then filtered and visualized using Cytoscape (version 3.10.1 [38]).

### Deposition of DNA sequence data

The draft genome sequences of the strains DT45^T^, DT32, DT55, DT59, DT194, DT15, DT42, DT43, DT44, DT56, DT60, DT69 and DT190 have been deposited in the DDBJ/ENA/GenBank under the accession numbers listed in Table 1. The versions described in this article are versions JBLLDX010000000, JBLLDW010000000, JBLLDT010000000, JBLLDU010000000, JBLLDV010000000, JBNCKJ010000000, JBNCKI010000000, JBNCKR010000000, JBNCKS010000000, JBNCOC010000000, JBNCNN010000000, JBNBLK010000000 and JBNCNM010000000 and are linked to the NCBI BioProject accession numbers PRJNA1199750, PRJNA1211321 and PRJNA1249248, with BioSample accession numbers SAMN45887308, SAMN45892500, SAMN45892501, SAMN45892502, SAMN45892503, SAMN46229778, SAMN46229779, SAMN46229770, SAMN46229769, SAMN47951508, SAMN47951523, SAMN47884541 and SAMN47951525. The 16S rRNA gene sequences of the strains are available in the GenBank database (PV139158, PV139159, PV139160, PV139204, PV139205, PV164011, PV164012, PV164014, PV164015, PV164016, PV164017, PV164018 and PV164024).

## Results and discussion

**Fig. 1.**
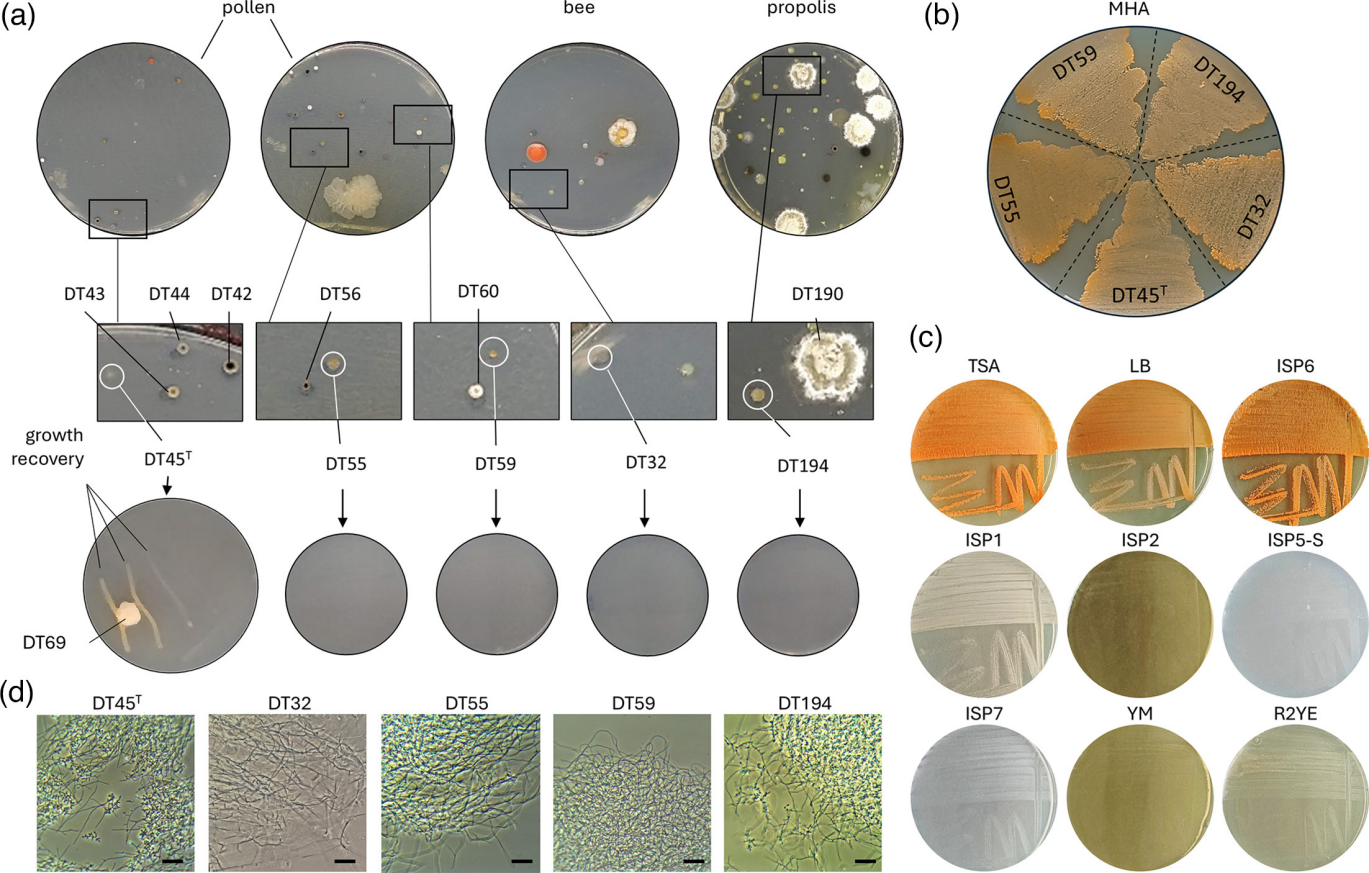
Identification of beehive-associated auxotrophic actinobacterial isolates. (a) Top panels: original ISP5-S agar plates of sample isolation, showing DT45^T^ and four other isolates (white circle, middle panels) that fail to grow when transferred to a new ISP5-S agar plate. Neighbouring strains from the original plate are indicated. Bottom panels: growth recovery of strain DT45^T^ only at proximity of a contaminant bacterial colony (DT69). (b) Normal growth on the rich MHA medium of the five strains, unable to grow alone on ISP5-S. (c) Growth capacity of DT45^T^ on various solid culture media. (d) Light microscopy pictures of DT45^T^ and related auxotrophic isolates grown in liquid MHB. Bar ~10 µM.

A bioprospection study was conducted in May 2021 to isolate actinomycetes from various sources within a beehive, including pollen, propolis, wax, honey and the surface of a honeybee (*A. mellifera*). During the subculturing, strains DT45^T^, DT55, DT59 (pollen), DT32 (bee) and DT194 (propolis) were unable to grow on the new agar plate as axenic cultures (Fig. 1a). As we used the same medium (ISP5-S) as initially used for selective isolation from environmental samples, this lack of growth suggested a potential dependence on microbial interactions on the original plates from which colonies were taken. This hypothesis was confirmed by the observation that one strain, DT45^T^, resumed growth when streaked near a bacterial colony (strain DT69) that had contaminated the plate (Fig. 1a). We rescued strain DT45^T^ and the four other strains (DT32, DT55, DT59 and DT194) that similarly failed to resume growth on ISP5-S by transferring the remaining part of the colony on the original plates to the rich and complex MHA medium, which was revealed to contain enough essential nutrients and/or appropriate conditions for their growth (Fig. 1b). When inoculated on other media, DT45^T^ exhibited various degrees of growth, ranging from none or poor (R2YE, YM, ISP2 and ISP7) to abundant (TSA, LB, ISP6 and ISP1). Growth on TSA, LB and ISP6 coincided with the intracellular production of an orange pigment (Fig. 1c). Observations under light microscopy revealed an unbranched mycelial morphology, indicating that the five isolated auxotrophic strains are likely filamentous actinomycetes (Fig. 1d).

**Fig. 2.**
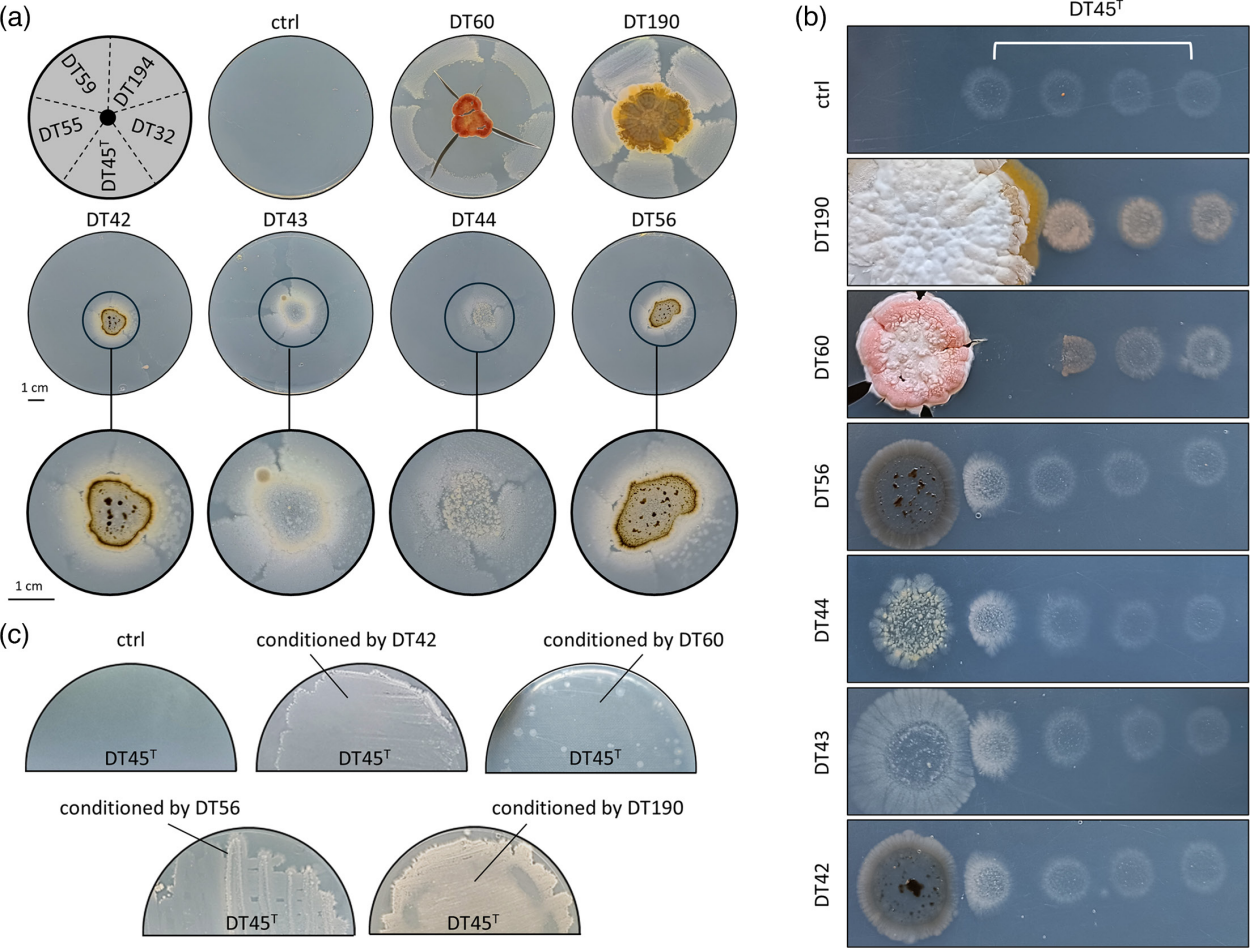
Cross-feeding-dependent growth of DT45^T^ and four additional strains unable to grow independently on ISP5-S medium. (a) Growth recovery of DT45^T^ and the four other isolates on ISP5-S agar plates when co-cultured with their neighbouring strains. Pictures were taken after 1 month of incubation at 28 °C. (b) Growth-promoting effect observed when neighbouring isolates are spotted near DT45^T^ on ISP5-S agar plate. Pictures taken after 1 month of growth. (c) Recovery of DT45^T^ growth on ISP5-S agar plate conditioned by neighbouring strains.

To determine whether the growth of these five rescued strains might depend on cross-feeding, we co-inoculated them onto ISP5-S medium alongside their nearest neighbouring strain(s) from the original plate of isolation (Fig. 1a). According to genome-based phylogenomics, four of these neighbouring strains (DT42, DT43, DT44 and DT56) belong to the genus *Micromonospora*, while strains DT60 and DT190 are members of the *Actinomadura* and *Streptomyces* genera, respectively (Fig. S1, available in the online Supplementary Material). As previously observed, none of the five isolates grew on ISP5-S medium alone; however, when a neighbouring strain was co-inoculated at the centre of the plate, growth was significantly improved (Fig. 2a). The most pronounced effect was observed with *Streptomyces* sp. DT190, where full growth recovery occurred even at the farthest distance from the rescuing strain. In contrast, *Actinomadura* sp. DT60, which produces antimicrobials on ISP5-S medium (possibly mathermycin/cinnamycin and/or madurastatin, based on genome mining), fully inhibited the growth of the auxotrophic strains in its vicinity, while growth was rescued only at a greater distance. Lastly, a growth-promoting effect was also seen when the four *Micromonospora* strains were inoculated at the centre of the plate, though growth was limited to the areas directly in contact with the rescuing strains (Fig. 2a, middle and bottom panels).

Interspecific growth stimulation was similarly observed when strain DT45^T^ was inoculated as spots at various distances from the rescuing strain (Fig. 2b). The growth-promoting effect also varied according to the strain in contact with strain DT45^T^ and the distance between inoculated spots. Strain DT190, which showed the strongest growth on ISP5-S, was also the most effective at promoting strain DT45^T^ growth, even at the furthest distance. However, the vigorous growth of strain DT190 eventually outgrew the nearest DT45^T^ spot (Fig. 2b). In the case of strain DT60, its antimicrobial activity on ISP5-S completely inhibited the growth of the first DT45^T^ spot. The growth-stimulating effect was only marked on part of the second spot, where the antibiotic concentration was lower, but secreted factors remained sufficient to stimulate growth (Fig. 2b). For *Micromonospora* strains, the growth enhancement of strain DT45^T^ was only observed on the nearest spot, suggesting that the nutrients secreted by members of this genus have reduced diffusion into the agar and/or are either less abundant or less effective for cross-feeding. As further evidence of cross-feeding, we tested whether microbial growth on dishes of ISP5-S could alter the chemical properties of the medium to create favourable conditions for strain DT45^T^. For this purpose, strains DT42, DT190, DT56 and DT60 were cultured on ISP5-S covered by a cellophane disc, which was removed after 1 week of growth at 28 °C. As shown in Fig. 2(c), plates conditioned by strains DT42, DT190 and DT56 could improve the growth of strain DT45^T^. Expectedly, as strain DT60 produces antimicrobial compound(s) on ISP5-S (Fig. 2a), the growth of strain DT45^T^ was marginal and only visible as colonies mainly distributed at the periphery of the plate, where we assume the antibiotic to be produced in lower amounts (Fig. 2c).

**Fig. 3.**
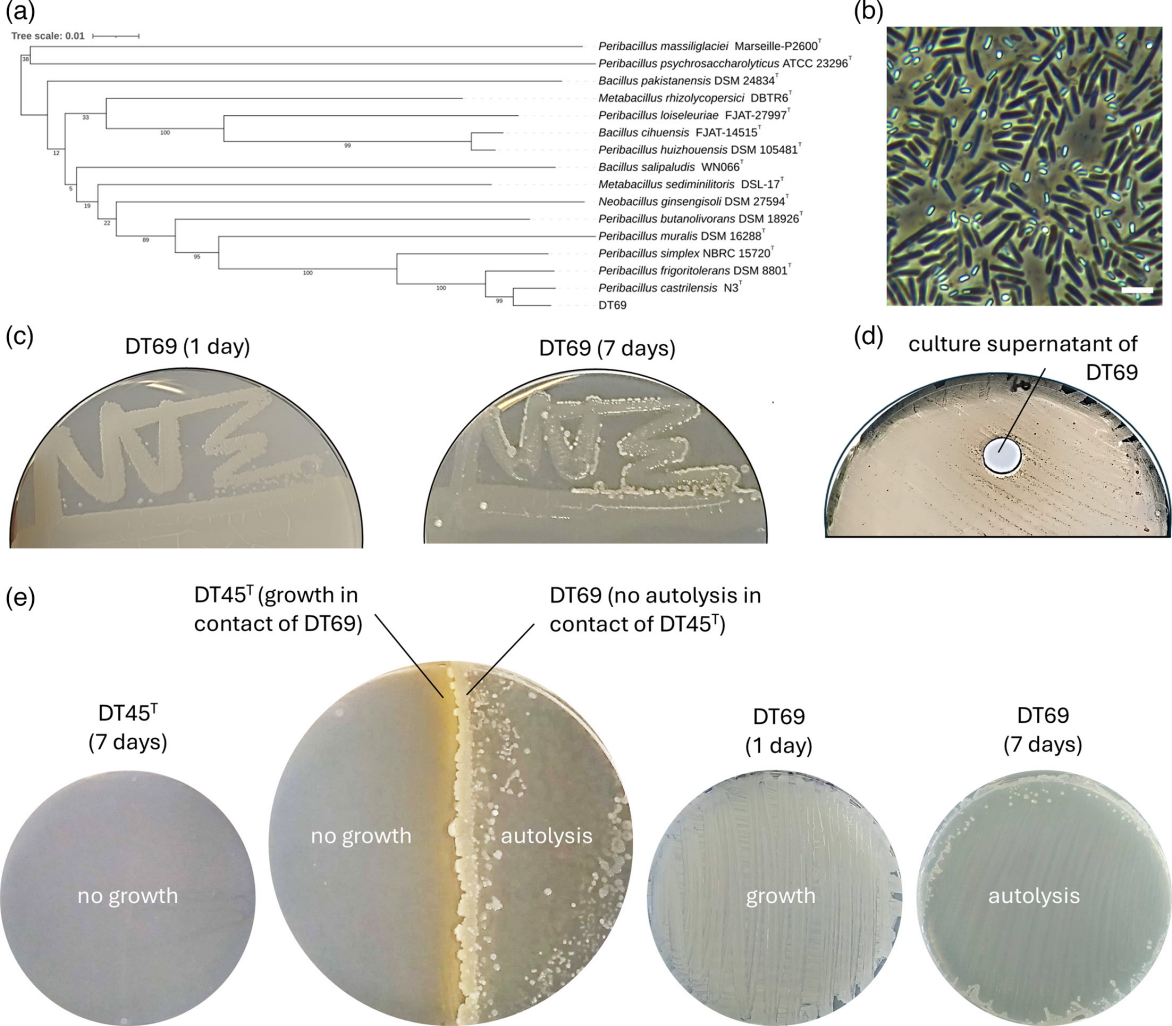
Phylogenomic analysis and morphology of strain DT69. (a) Phylogenomic tree inferred using FastME 2.1.6.1 [21] from GBDP distances calculated from whole-genome sequences, with data processed through the TYGS [18] and visualized using the iTOL v6 [22]. Branch lengths are scaled in terms of GBDP distance formula d_5_. GBDP pseudo-bootstrap support values (numbers below branches) are based on 100 replicates, with an average branch support of 62.4%. The tree was rooted at the midpoint and highlights the most closely related type strains to strain DT69. (b) Phase contrast microscopy image of strain DT69. Bar ~5 µm. (c) Growth dynamics of strain DT69 on ISP5-S agar plate, with autolysis observed after 7 days. (d) Growth stimulation of strain DT45^T^ by the ISP5-S culture supernatant from strain DT69, the contaminating strain that initially rescued DT45^T^ growth. (e) The physical contact between DT45^T^ and DT69 is mutually beneficial.

Finally, we investigated if strain DT69 – the contaminant strain that originally rescued growth of strain DT45^T^ (Fig. 1a) – was also able to stimulate DT45^T^ growth through cross-feeding (Fig. 3). Phylogenomic analysis of the whole genome by dDDH using the TYGS [18] database identified strain DT69 as belonging to the *Peribacillus castrilensis* species (84.8% dDDH) (Fig. 3a). Strain DT69 displays a rod-shaped morphology typical of *Peribacillus* species and produces spores when cultured on liquid MHB (Fig. 3b). Remarkably, strain DT69 undergoes extensive autolysis after 7 days when grown on ISP5-S, which could release the nutrients lacking for strain DT45^T^ (Fig. 3c). This hypothesis was confirmed as the addition of the ISP5-S culture supernatant from strain DT69 strongly stimulated the growth of DT45^T^ (Fig. 3d). Furthermore, physical contact between strain DT45^T^ and strain DT69 appears mutually beneficial: strain DT69 supports the growth and development of strain DT45^T^, as evidenced by the orange pigmentation visible only in the contact zone between the two species (Fig. 3e). In return, strain DT69 does not undergo autolysis when in the physical contact zone with strain DT45^T^ (Fig. 3e).

### Phylogenomic analyses of DT45^T^

The genomes of strain DT45^T^ and the four other auxotrophic strains, DT32, DT55, DT59 and DT194, were extracted, sequenced, annotated and uploaded for phylogenomic comparison using both 16S rRNA gene and whole-genome sequences against all genomes available in the TYGS database. Comparisons of 16S rRNA gene sequences revealed that the five strains belong to the same species cluster (Fig. S2). A blastn search of the 16S rRNA gene sequence against the genomes of the closest type strains revealed 16S rRNA gene sequence identities of 98.6 and 98.2% with *Micromonospora pisi* DSM 45175^T^ and *Polymorphospora rubra* NBRC101157^T^, respectively. The PCR amplified the 1,034 bp 16S rRNA gene sequence of strain DT45ᵀ, which was found to be identical to the corresponding sequences obtained from whole-genome sequencing.

**Fig. 4.**
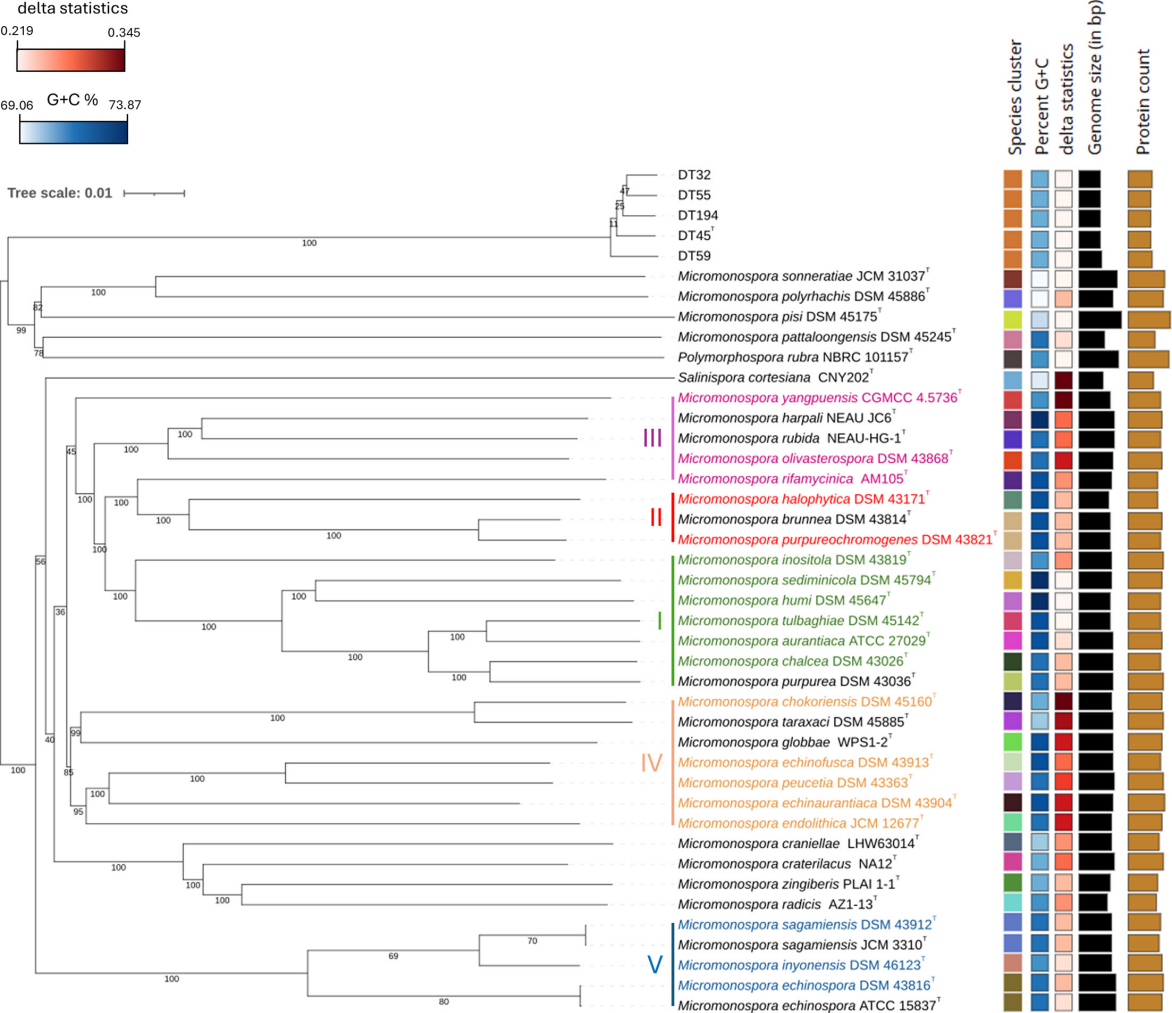
Phylogenomic tree based on whole-genome sequences of isolate DT45^T^ and related isolates. The tree was inferred using FastME 2.1.6.1 [21] from GBDP distances calculated from whole-genome sequences. Data were processed through the TYGS [18] and visualized using the iTOL v6 [22]. Branch lengths are scaled according to the GBDP distance formula d_5_. GBDP pseudo-bootstrap support values (numbers below branches) were calculated from 100 replicates, with an average support of 85.9%. The tree is rooted at the midpoint and illustrates the *Micromonospora* spp. most closely related to the isolates described. Label colours represent the five clades within the *Micromonospora* genus according to Carro *et al*. [39]. The genome size is between 4,549,447 and 8,739,867 bp. The protein count is between 4,192 and 7,627.

The whole-genome-based phylogenomic analysis provided clearer resolution, confirming the separation of the five beehive isolates into a distinct species cluster (Fig. 4). Interestingly, these five auxotrophic strains do not align with any of the five recognized clades within the *Micromonospora* genus as defined by Carro *et al*. [39]. Instead, they form a distinct monophyletic group, strongly supported by a 100% bootstrap value and dDDH values ranging from 85.0 to 91.5%. Interestingly, their closest relatives are type strains whose classification within the *Micromonospora* genus is currently questioned (Carro L., personal communication). These include *Micromonospora sonneratiae* JCM 31037^T^, *Micromonospora polyrhachis* DSM 45886^T^, *Mi. pisi* DSM 45175^T^ and *Micromonospora pattaloongensis* DSM 45245^T^, whose position within the phylogenomic tree also supports their distance from other members of the genus. The pairwise dDDH values between beehive isolates and their closest relatives range from 21.8 (*Mi. pisi* DSM 45175^T^) to 22.1% (*Mi. sonneratiae* JCM 31037^T^ and *Mi. pattaloongensis* DSM 45245^T^), which is significantly below the 70% threshold established for species delineation [40]. Similar low pairwise dDDH values are observed with the closest relatives that belong to non-*Micromonospora* genera, i.e. 21.3% for *Salinispora cortesiana* CNY202^T^ and 22.0% for *P. rubra* NBRC101157^T^, suggesting that DT45^T^ may be a representative of a novel bacterial genus within the *Micromonosporaceae* family.

Another particularly striking difference between the beehive isolates and strains within the *Micromonospora* genus and other closely related genera is their genome size (Fig. 4). The complete genome of strain DT45^T^ consists of a single chromosome, measuring 4,607,559 bp and encoding 4,227 protein-coding genes, which is significantly smaller than the 6 to 8 Mbp average genome size typically observed in *Micromonospora* species [39]. Similarly, the other four DT strains display comparable genomic characteristics (Table S1), with an average genome size of ~4.6 Mbp. The genome of strain DT45^T^ has a G+C content of around 71.6 mol%, aligning with values commonly observed in *Micromonosporaceae*.

**Fig. 5.**
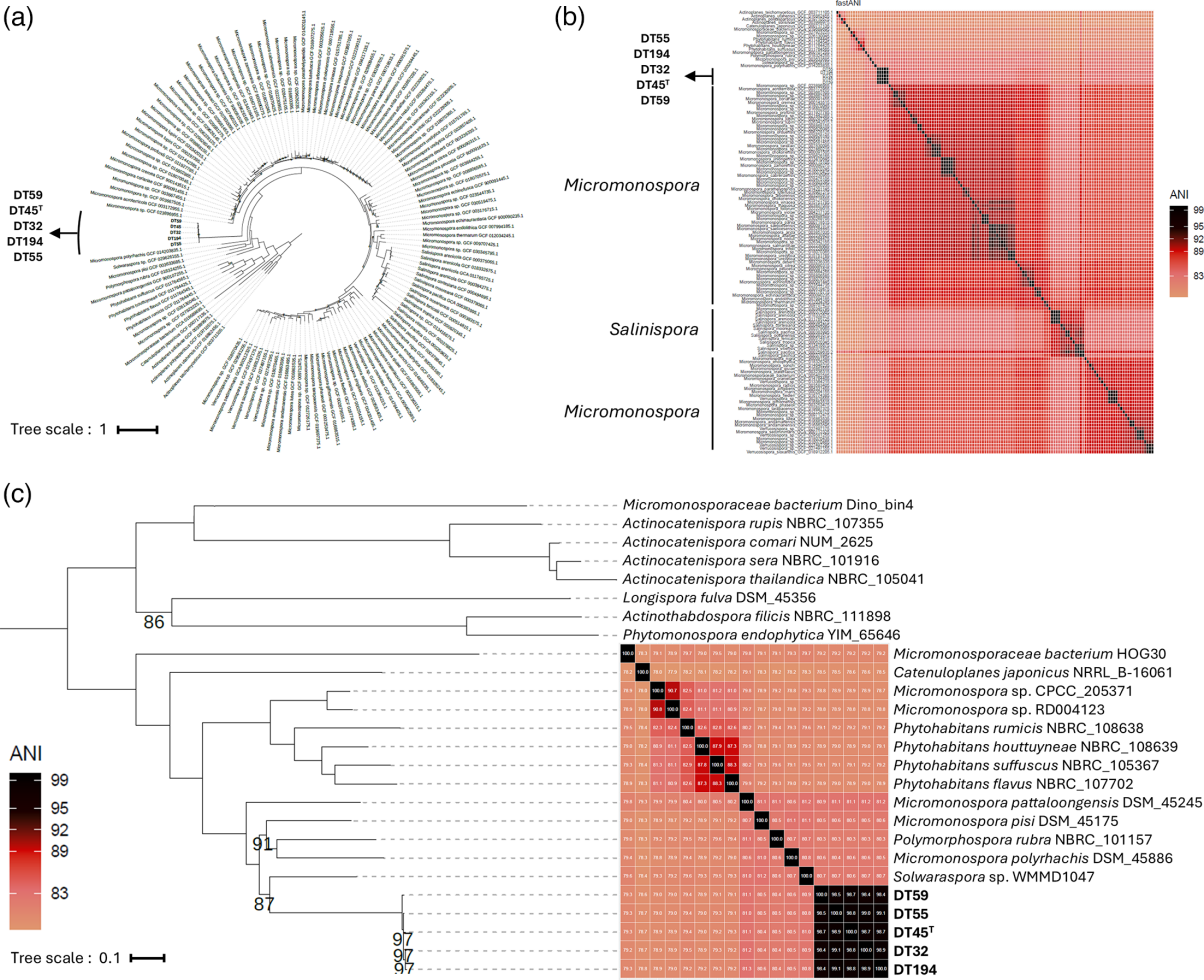
Phylogenomic and ANI analysis of strain DT45^T^ and related type strains. A large (**a and b**) and a focused (**c**) dataset of 133 and 26 genomes, respectively, were used in the Orthodox and Phylogenomic workflows of GENERA [23] to infer phylogenomic trees constructed by RaxML [32] using the PROTGAMMALFG model and in the ANI workflow of GENERA [23] to infer heatmaps using fastANI [24] and ggplot2. The high-resolution figures are provided in Figs S3–S6.

To further assess the cluster delineation of our five auxotrophic beehive isolates, we performed a phylogenomic analysis based on a complete set of *Actinomycetota* genomes (Fig. 5). A preliminary phylogenomic analysis based on 459 genomes (available upon request) resulted in the creation of two datasets: a large phylogenomic dataset consisting of 133 genomes (Figs 5a, b, S3 and S4, respectively) and a focused analysis with 26 genomes (Figs 5c, S5 and S6). For each of these analyses, a new orthology analysis was performed to increase the number of core genes. In both cases, ANI comparisons (Figs 5b-large, c-focused, S4 and S6) were conducted using fastANI [24] through the GENERA [23] workflow. This additional whole-genome-based phylogenomic analysis confirmed the separation of the five beehive isolates into a distinct species cluster. Focused ANI heatmaps (Figs 5c and S6) were generated to visually represent the relationships between these genomes, further confirming that our strains appear to belong to a distinct group separate from the *Micromonospora* genus, with the exception of *Mi. polyrhachis* DSM 45886^T^, *Mi. pisi* DSM 45175^T^ and *Mi. pattaloongensis* DSM 45245^T^, whose classification within the *Micromonospora* genus is currently debated (as discussed above). Furthermore, the closely related strain, with an ANI percentage of 81%, belongs to the ‘*Solwaraspora*’ genus (*Solwaraspora* sp. WMMD1047^T^), a described genus from a marine environment whose name has not been validated [41]. In order to determine the position of the isolates with respect to the type species of all genera within the family *Micromonosporaceae*, a 16S rRNA gene phylogenetic tree was performed, which confirms its position as an independent genus within the family (Fig. S7), more closely related to the *Micromonospora*, *Plantactinospora*, *Polymorphospora*, *Salinispora* and *Rhizomonospora* genera.

Finally, we applied the Protologger tool to characterize the ecological distribution and habitat preferences of our isolates and found no metagenome-assembled genomes matching the genome of the DT45^T^ strain. In addition, testing against 1,000 amplicon sequences from different environments yielded detection percentages: our species was detected in 62.8% of rhizosphere metagenomes, 42.5% of soil metagenomes and 24.8% of plant metagenomes, with less than 15% detected in other environments and only 1.4% of insect gut metagenomes (Table S2). In all those environments, strain DT45^T^ showed values of relative abundance lower than 0.25%, with maximum relative abundances for soil (0.24%), plant (0.21%) and rhizosphere (0.14%). These results may in part explain why this genus has not been isolated previously.

### Phenotypic characterization

**Fig. 6.**
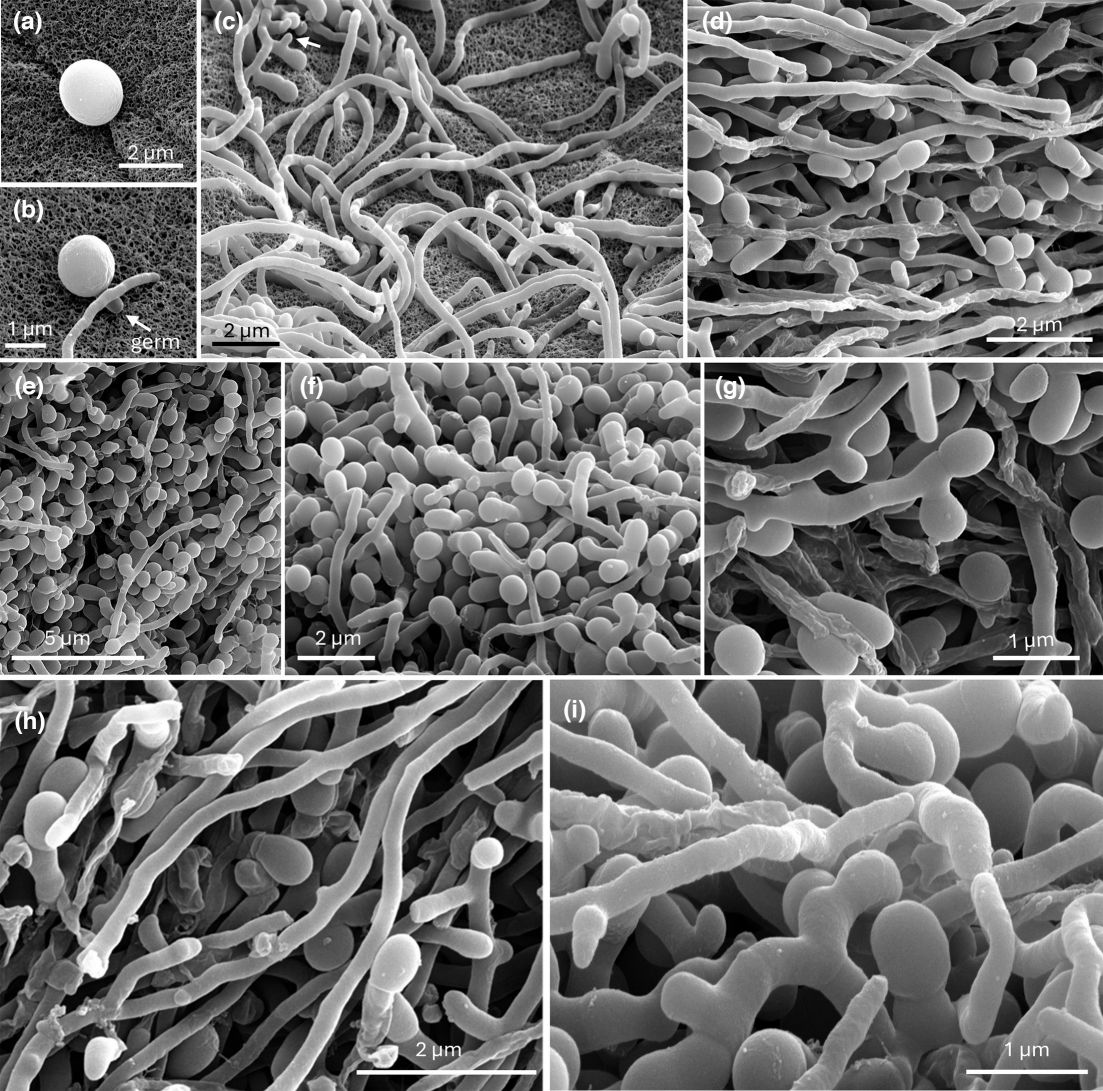
Cryo-scanning electron micrographs of strain DT45^T^ grown on MHA at 28 °C for 1 week. For a detailed description of the different panels, refer to the main text.

Scanning electron microscopy provided detailed visualization of the DT45^T^ life cycle, including spore germination (Fig. 6a, b), vegetative mycelium growth (Fig. 6c) and various stages of sessile monospore formation (Fig. 6d–i). Fig. 6(a) depicts a swollen spore due to hydration, just before the emergence of a germination tube (Fig. 6b). Consistent with light microscopy findings, strain DT45^T^ predominantly forms unbranched filaments (Fig. 6c), contrasting with the monopodially branched mycelium typical of the *Micromonospora* genus [42]. The first signs of sporulation are marked by small protuberances on the surface of the vegetative mycelium (arrows in Fig. 6c, d). Single spore formation occurs directly on the substrate mycelium, without the development of aerial hyphae. The various stages in the sporulation process are illustrated in Fig. 6(d–i). Sessile spores have a smooth, unornamented surface. At the edge of the confluent lawn on the agar plate, a greater density of spores was observed, with spores emerging from multiple buds along single filaments (Fig. 6e, f).

### Chemotaxonomic characterization

**Fig. 7.**
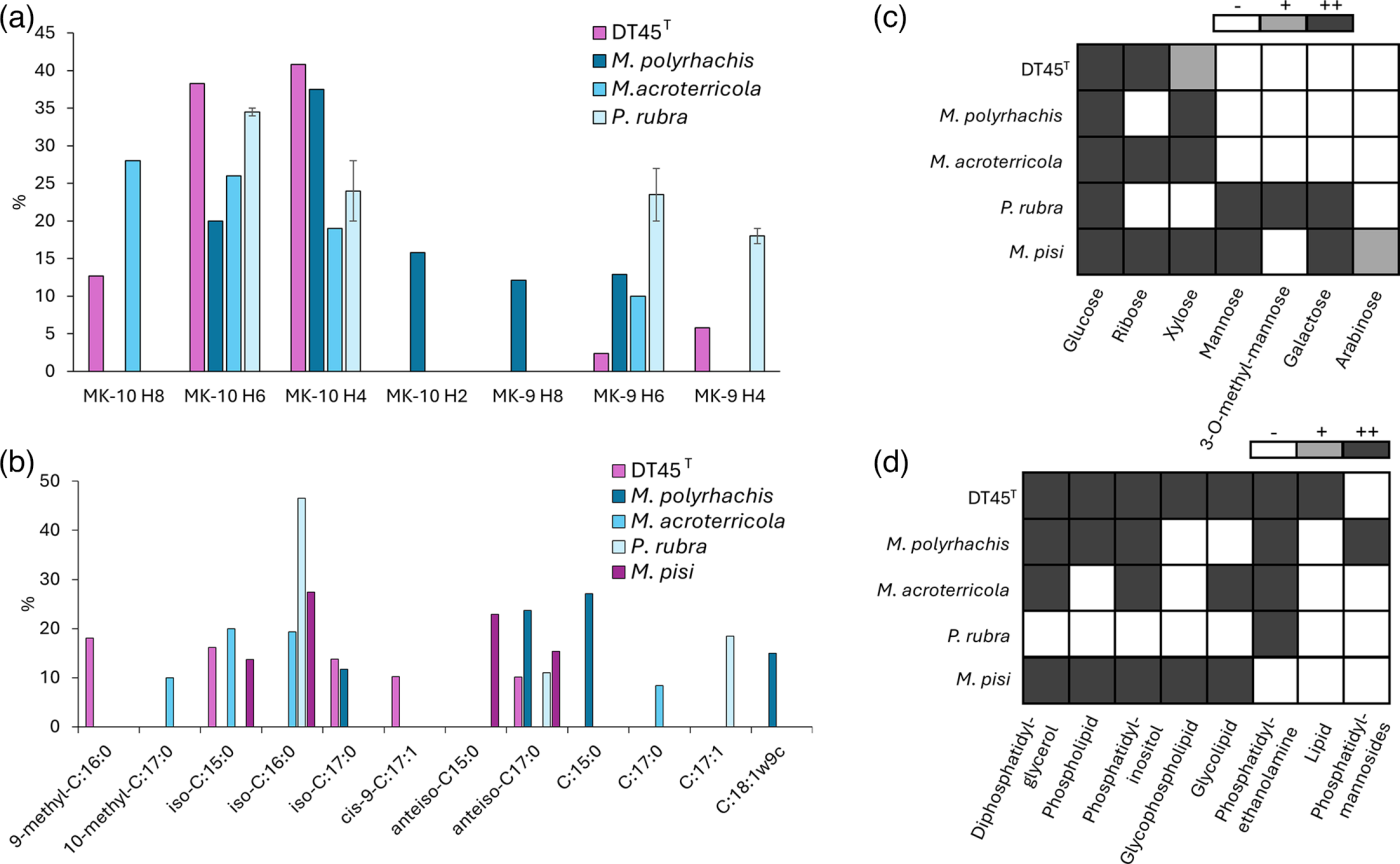
Chemotaxonomic characterization of strain DT45^T^. (a) Respiratory menaquinones. (b) Major fatty acids. (c) Whole-cell sugars. (d) Polar lipids. Note: chemotaxonomy data for the four phylogenetically closest species are included for comparison [49,52].

Chemotaxonomy analyses were conducted by the Identification Service of DSMZ (Braunschweig, Germany). HPLC-DAD-MS analysis detected the following respiratory menaquinones (MK): MK-10 H_4_ (40.8%), MK-10 H_6_ (38.3%), MK-10 H_8_ (12.7%), MK-9 H_4_ (5.8%) and MK-9 H_6_ (2.4%) (Fig. 7a). The major fatty acid was 9-methyl-C_16:0_ (18.1%), which has not been previously described for members of the genus *Micromonospora*, where iso-C_16:0_ is commonly found [39]. This is a major difference, as this fatty acid has been described in other members of the family *Micromonosporaceae*, but not for any *Micromonospora* species. Other major fatty acids included iso-C_15:0_ (16.2%), iso-C_17:0_ (13.8%), cis-9-C_17:1_ (10.2%) and anteiso-C_17:0_ (10.1%) (Fig. 7b). The whole-cell hydrolysate revealed glucose and ribose as the major sugars, with minor amounts of xylose (Fig. 7c). The most abundant polar lipids of strain DT45^T^ were diphosphatidylglycerol, phosphatidylethanolamine, phosphatidylinositol and glycophospholipids (Figs 7d and S8). No polyamines were detected in the analysed samples. Finally, the peptidoglycan type was concluded to be A1*γ*′ *meso*-Dpm-direct, as is typically expected for members of the *Micromonospora* genus.

### Physiological characterization

**Fig. 8.**
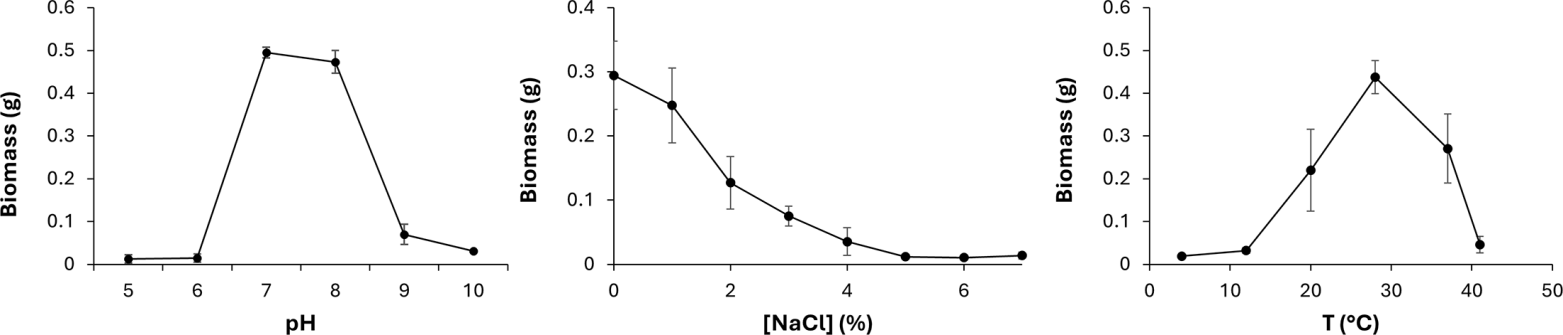
pH, salinity and temperature tolerance of strain DT45 ^T^. Biomass values are for 25 ml of culture medium.

Strain DT45^T^ grew in liquid MHB at temperatures ranging from ~15 to 37 °C, pH 6.5–9 and in the presence of 1–4% NaCl, with optimal biomass accumulation observed at 28 °C and a pH of 7–8 (Fig. 8).

**Fig. 9.**
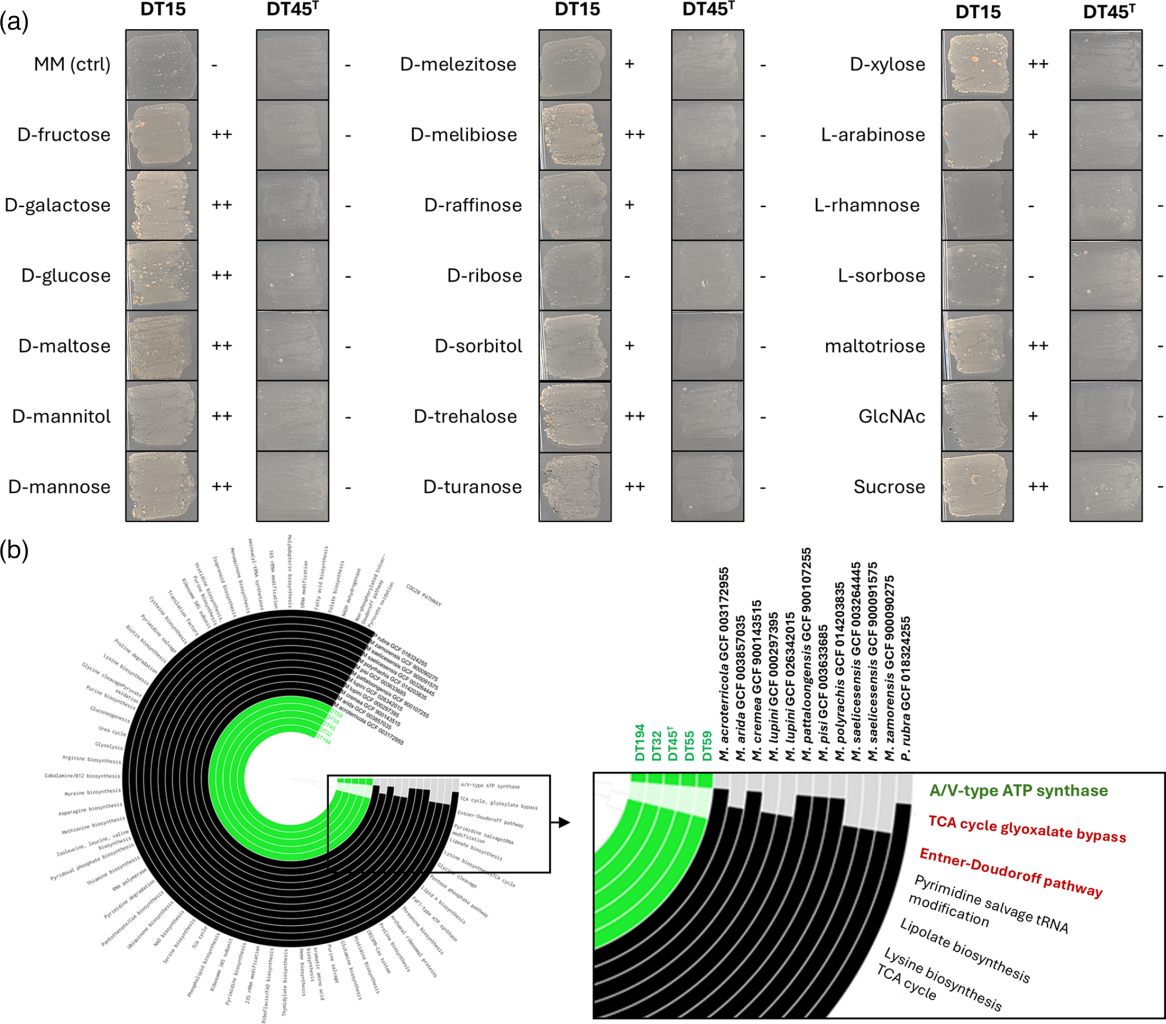
Assessment of the level of auxotrophy of strain DT45^T^ for carbon sources. (a) Growth of strain DT45^T^ on different carbon sources. Images of strains DT15 and DT45^T^ grown for 7 days on MM supplemented with different carbon sources at 1% (v/v). Symbol: -, no growth; +, moderate growth; ++, good growth. (b) Pangenomics of DT45^T^ and closest *Micromonospora* strains. Note the gain of the A/V-type ATP synthase pathway and the loss of the ED pathway and glyoxylate bypass. The high-resolution full-size figure generated using Anvi’O v8 [34] is provided as Fig. S9.

To initially assess the level of auxotrophy of strain DT45^T^, the mycelium from a 1-week preculture in MHB was used to inoculate MM plates supplemented with 20 different carbon sources (Fig. 9a). Unexpectedly, strain DT45^T^ failed to grow on any of these substrates, whereas the non-auxotrophic control strain, *Micromonospora* sp. DT15, demonstrated growth on most of the tested sugars (Fig. 9a). To gain deeper insights into the genomic features of strain DT45^T^ and its related strains (DT32, DT55, DT59 and DT194) that may explain such a level of auxotrophy for carbon sources, we conducted a pangenomic analysis, comparing their genomes with those of the 12 most phylogenetically related strains (Fig. 9b, see Fig. S9 for the high-resolution full-size figure of the pangenomic analysis). Our analysis revealed the absence of the glyoxylate shunt and the classical Entner–Doudoroff (ED) pathway in the auxotrophic strains. The absence of the glyoxylate bypass suggests a limited capacity of these strains to grow on carbon sources such as acetate or fatty acids. This loss forces the strain to rely on the full TCA cycle, resulting in more carbon lost as CO₂ [43], and necessitates the synthesis of anabolic precursors through more energy-intensive routes, reducing biomass generation efficiency. The ED pathway is essential for glucose catabolism, offering an alternative to glycolysis (Embden–Meyerhof–Parnas, EMP) and the pentose phosphate pathway (PPP). This pathway converts glucose to pyruvate while generating NADPH, which is essential for counteracting oxidative stress [44]. In *Pseudomonas putida*, the absence of the ED pathway reduces NADPH availability, impairing the bacterium’s ability to defend against oxidative stress [45].

The loss of the ED pathway forces the use of more complex or less efficient metabolic routes (EMP, PPP) for carbon source utilization. However, our strains possess genes for the non-phosphorylative variant of the ED pathway. In this non-phosphorylative route, glucose is oxidized to gluconate, dehydrated to 2-keto-3-deoxygluconate and then cleaved into pyruvate and glyceraldehyde. This variant, observed in certain archaea like *Picrophilus torridus*, provides an alternative mechanism for glucose degradation [46]. In contrast, all other tested strains in the pangenomic analysis possess both the traditional phosphorylative ED pathway and the non-phosphorylative variant, highlighting a unique metabolic feature of our strains that could influence their NADPH balance and oxidative stress response.

Additionally, genes linked to the A/V-type ATP synthase pathway were uniquely identified in our five auxotrophic strains. While F-type ATP synthases are primarily found in bacteria, A/V-type ATP synthases are typically associated with archaea [47]. Although A/V-type ATP synthases are predominantly found in archaea, some bacteria, such as *Thermus thermophilus*, are thought to have acquired them through horizontal gene transfer from hyperthermophilic archaea [47,48]. Both A- and F-type ATP synthases share the common function of ATP synthesis under physiological conditions [48]. The presence of A-type ATP synthases in our strains may indicate adaptations to specific environmental conditions or higher energy requirements, possibly as a consequence of the loss of the ED pathway. The combination of these genomic features could potentially lead to reduced metabolic efficiency, slower growth, limited metabolic flexibility and heightened sensitivity to environmental stresses, as observed in our five strains.

### Specialized metabolism

**Fig. 10.**
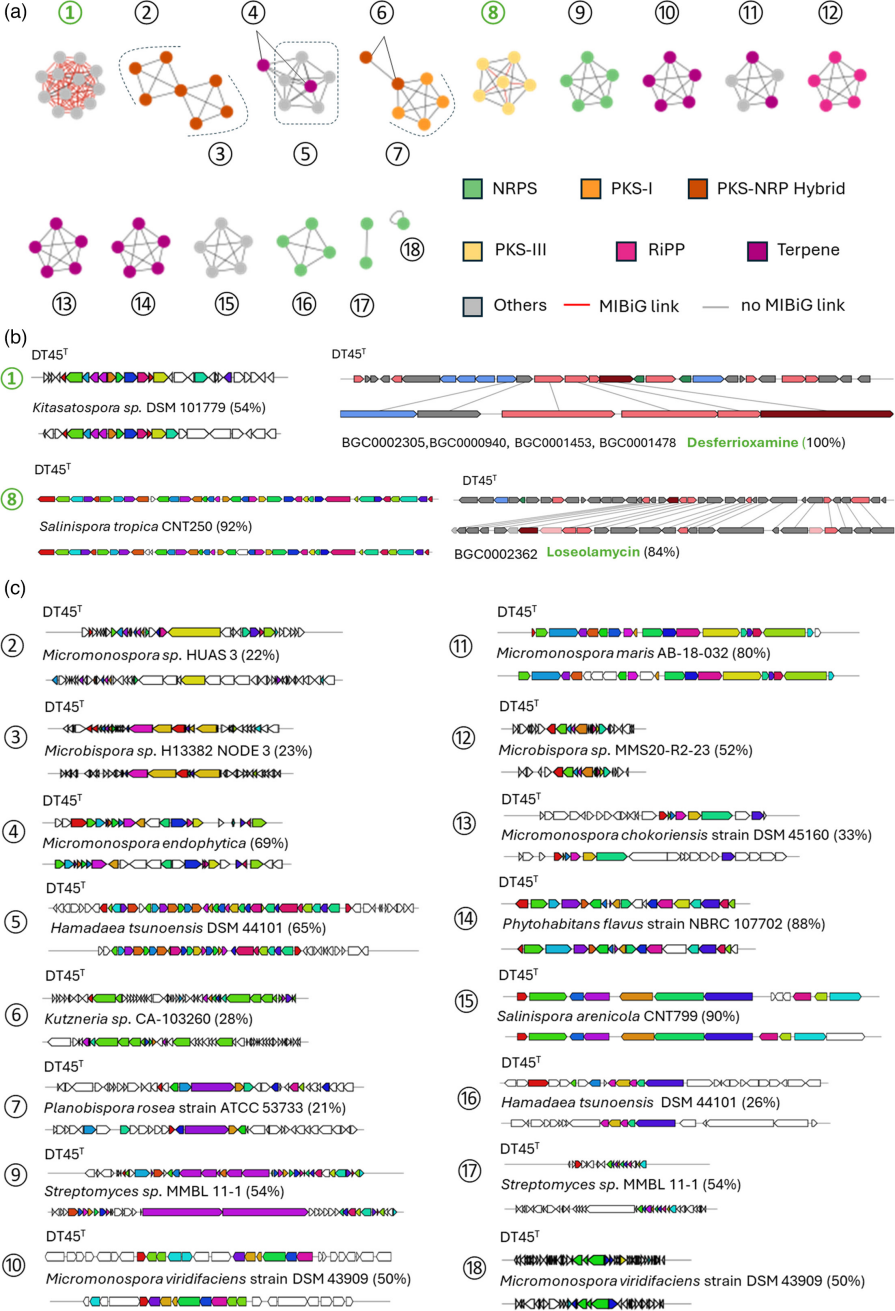
Identification of BGCs of strain DT45^T^ and related strains. (a) BiG-SCAPE visualization of networks of the predicted BGCs in strain DT45^T^ and related strains. (b) Gene organization of the two BGCs with high similarity to known BGCs of the MIBiG database. (c) Gene organization of the cryptic BGCs of strain DT45^T^ and related strains and comparison with their respective most similar BGCs retrieved from the antiSMASH database.

The genetic potential of strain DT45^T^ and related strains to produce specialized (secondary) metabolites was evaluated by mining their genomes for BGC with the antiSMASH software [35]. The sequence similarity networks of the predicted BGCs were subsequently generated and compared to the minimum information about BGC (MIBiG v3.0 [36]) repository using the Biosynthetic Gene Similarity Clustering and Prospecting Engine software (BiG-SCAPE v1.1.5 [37]). This analysis identified a total of 18 BGCs distributed across different BGC categories, including NRPS (4), PKS-NRP hybrid (3), PKS-I (1), PKS-III (1), terpene (4) and other (2) (Fig. 10 and Table 2). Eleven BGCs were conserved across the five strains, indicating that, while these strains belong to the same species, part of their specialized metabolism is unique to certain strains. This divergence may possibly result from adaptations to the beehive environment from which they were isolated (pollen, bees and propolis). A manual inspection of BGC’s synteny and genes/proteins homology with their closest known BGCs revealed that natural products could be reliably linked to only two BGCs (Fig. 10b). These included the desferrioxamine siderophores, which are part of the core metabolome of actinobacterial species, and the antibacterial and phytotoxic loseolamycin compounds. The presence of a BGC for siderophore biosynthesis and uptake of ferri-siderophores suggests that the lack of growth of strain DT45^T^ is unlikely to be due to the inability to acquire iron from the cultivation medium. This possibility was definitively ruled out, as the supply of desferrioxamine B to the ISP5 medium could not restore the growth of strain DT45^T^ (not shown).

**Table 2.** List of BGCs identified in the five DT strains

BGC	BGC type	Organism of closest BGC – (%)	MIBiG (%)	Compound	Occurrence
1	Others (siderophores)	*Kitasatospora* sp. DSM 101779 – (54)	BGC0002305 (100)	Desferrioxamines	5
2	PKS-NRP hybrid	*Micromonospora* sp. HUAS 3 – (22)	–	Unknown	3
3	PKS-NRP hybrid	*Microbispora* sp. H13382 – (23)	–	Unknown	4
4	Others	*Micromonospora endophytica* – (69)	–	Unknown	2
5	Others	*Hamadaea tsunoensis* DSM 44101 – (65)	–	Unknown	5
6	PKS-NRP hybrid	*Kutzneria* sp. CA-103260 – (28)	–	Unknown	2
7	PKS I	*Planobispora rosea* ATCC 53733 – (21)	–	Unknown	4
8	PKS III	*Salinospora tropica* CNT250 – (92)	BGC0002362 (84)	Loseolamycin	5
9	NRPS	*Streptomyces* sp. MMBL 11-1 – (54)	–	Unknown	5
10	Others	*Micromonospora viridifaciens* DSM 43909 – (50)	–	Unknown	5
11	Others	*Micromonospora maris* AB-18-032 – (80)	–	Unknown	5
12	RiPP	*Microbispora* sp. MMS20-R2-23 – (52)	–	Unknown	5
13	Others	*Micromonospora chokoriensis* DSM 45160 – (33)	–	Unknown	5
14	Others	*Phytohabitans flavus* NBRX 107702 – (88)	–	Unknown	5
15	Others	*Salinispora arenicola* CNT799 – (90)	–	Unknown	5
16	NRPS	*Hamadaea tsunoensis* DSM 44101 – (26)	–	Unknown	4
17	NRPS	*Streptomyces* sp. MMBL 11-1 – (54)	–	Unknown	2
18	NRPS	*Micromonospora viridifaciens* DSM 43909 – (50)	–	Unknown	1

%, percentage of similarity with either their closest match or with the closest BGC of the MIBiG repository. Occurrence refers to the number of strains (out of five) in which a specific BGC was identified.

The other 16 BGCs are therefore defined as cryptic, meaning that a natural product cannot be reliably associated with the genetic material (Fig. 10c and Table 2). Interestingly, the closest homologues of these cryptic BGCs are identified in different actinobacterial genera, including *Micromonospora* (6 BGCs), *Hamadaea* (2 BGCs), *Streptomyces* (2 BGCs), *Microbispora* (2 BGCs), *Salinispora* (1 BGC), *Phytohabitans* (1 BGC), *Kutzneria* (1 BGC) and *Planobispora* (1 BGC). The observation that the closest BGCs are associated with eight different actinobacterial genera suggests that this genetic material was likely acquired through multiple horizontal gene transfer events followed by extensive mutagenesis and gene reorganization. While we cannot entirely rule out the possibility that some of these BGCs were generated within these strains, their endemic distribution, limited exclusively to the studied beehive environment, strongly supports horizontal gene transfer as the more plausible explanation for their origin. The presence of 12 BGCs whose closest homologs do not belong to *Micromonospora* species further confirms that strain DT45^T^ and related strains could be the representative members of a novel bacterial genus.

## Conclusions

In conclusion, our study underscores the complexity of microbial diversity and the critical role of microbial interactions in shaping the growth and survival of micro-organisms, particularly those that remain unculturable under standard laboratory conditions. The discovery of a novel lineage within the *Micromonosporaceae* family, *M. conviva*, highlights the ecological specialization of these micro-organisms within their natural environments. The genomic, physiological and chemotaxonomic data collectively demonstrate the unique metabolic dependencies of these isolates, offering insights into their evolutionary divergence and the mechanisms underlying their inability to survive outside their native microbial communities.

Beyond its taxonomic significance, this study raises broader questions about the ecological roles of auxotrophic bacteria in complex microbial communities. Future research should investigate the specific metabolic exchanges that sustain *M. conviva*, its potential contribution to the beehive microbiome and whether similar cross-feeding dependencies exist in other environments. Understanding these interactions could provide valuable insights into microbial cooperation, natural product biosynthesis and strategies for cultivating previously unculturable micro-organisms.

## Protologue

### Description of *Melissospora* gen. nov.

*Melissospora* [Me.lis.so.spo’ra. Gr. fem. n. *melissa*, bee; Gr. fem. n. *spora*, seed; N.L. fem. n. *Melissospora*, seed of the (honey) bee].

Colonies are raised and folded, circular and with irregular edges on MHA. Cells are aerobic, Gram-positive, non-motile, filamentous and spore-forming (0.5–1 µm in diameter). Cell-wall peptidoglycan contains *meso*-DAP, peptidoglycan type A1*γ*′. Cell-wall sugars include glucose and ribose. The major fatty acids are branched-chain acids, including 9-methyl-C_16:0_, iso-C_15:0_, iso-C_17:0_ and cis-9-C_17:1_. The main menaquinones are MK-10 (H4) and MK-10 (H6). The polar lipid profile includes phosphatidylethanolamine. This genus belongs to the family *Micromonosporaceae*, being most closely related to the genera *Micromonospora*, *Polymorphospora*, *Salinispora* and *Phytohabitans*. The DNA G+C content is ~71.6 mol%. The type species is *M. conviva*.

### Description of *Melissospora conviva* sp. nov.

*Melissospora conviva* (con.vi’va. L. fem. n. *conviva*, a commensal).

Aerobic, Gram-stain-positive actinomycete that forms non-motile, single spores (0.5–1 µm) with smooth surfaces on extensively branched, non-fragmenting substrate hyphae. Colonies are raised and folded; light orange coloured with no aerial hyphae on MHA. Grows from pH 6.0 to 9.0 (optimum 7.0–8.0), from 15 to 37 °C (optimum 28 °C) and in the presence of up to 1% of NaCl (w/v). Grows well in TSA, LB, ISP6 and ISP1 media and poorly on R2YE, ISP2 and ISP7. Strains of this species were unable to grow onto ISP5-S medium without co-inoculation with other strains. d-fructose, d-galactose, d-glucose, d-maltose, d-mannitol, d-mannose, d-melezitose, d-melibiose, d-raffinose, d-ribose, d-sorbitol, d-trehalose, d-turanose, d-xylose, l-arabinose, l-rhamnose, l-sorbose, malotriose, GlucNac and sucrose were not used as a sole carbon source. Peptidoglycan type is A1*γ*′ and contains *meso*-DAP. Cell-wall major sugars include glucose and ribose. Major fatty acids include 9-methyl-C_16:0_, iso-C_15:0_, iso-C_17:0_ and cis-9-C_17:1_. The main menaquinones are MK-10 (H_4_) and MK-10 (H_6_), and the main polar lipid profile includes diphosphatidylglycerol, phosphatidylethanolamine, phosphatidylinositol and glycophospholipids. Genome size is around 4.6 Mbp, and DNA G+C content is around 71.6 mol%.

The type strain, DT45^T^ (=DSM 117791^T^=LMG 33580^T^), was isolated from pollen of a beehive located in Comblain-au-Pont (Belgium). The DDBJ/EMBL/GenBank accession numbers for the 16S rRNA gene and genome sequences are PV139158 and JBLLDX000000000, respectively. Other strains of the species include DT32 (from bee), DT55 and DT59 (from pollen) and DT194 (from propolis). The type strain DT45^T^ has been deposited in the DSMZ and BCCM bacterial collections under strain designations DSM 117791 and LMG 33580, respectively.

## Supplementary material

10.1099/ijsem.0.006868 Uncited Supplementary Material 1.

## Abbreviations

ANI: average nucleotide identity
BGC: biosynthetic gene cluster
dDDH: digital DNA–DNA hybridization
ED: Entner–Doudoroff
EMP: Embden–Meyerhof–Parnas
FAME: fatty acid methyl ester
GBDP: genome blast distance phylogeny
HPLC-DAD-MS: HPLC coupled with diode-array detection and MS
ISP: International *Streptomyces* Project
iTOL: Interactive Tree of Life
LB: Luria–Bertani
MHA: Müller–Hinton agar
MHB: Müller–Hinton broth
MM: minimal medium
PPP: pentose phosphate pathway
TSA: tryptic soy agar
TYGS: type (strain) genome server
YM: Yeast-Malt
