# Supplementary material for: Melissospora conviva gen. nov., sp. nov., a novel actinobacterial genus isolated from beehive through cross-feeding interactions
Source: Int J Syst Evol Microbiol. 2025 Aug 11;75(8):006868. doi: 10.1099/ijsem.0.006868 (PMC12451618; doi:10.1099/ijsem.0.006868)
Supplement: Uncited Supplementary Material 1. [file ijsem-75-06868-s001.pdf]

Supplementary Material

Melissospora conviva gen. nov., sp. nov., a novel actinobacterial genus isolated from beehive through cross-feeding interactions

Déborah Tellatin[1], Luc Cornet[2], Valdes Snauwaert[3], Philippe Compère[4], Marc Ongena[5], Loïc Quinton[6], Nudzejma Stulanovic[1], Silvia Ribeiro Monteiro[1], Augustin Rigolet[7], Pierre Burguet[6], Petra Van Damme[3], Lorena Carro[8], and Sébastien Rigali[1,*]

[1]CIP, InBioS–Center for Protein Engineering, University of Liège, Institut de Chimie, Liège B-4000, Belgium;

[2]BCCM/ULC, InBioS–Molecular Diversity and Ecology of Cyanobacteria, University of Liège, Liège B-4000, Belgium;

[3]iR.I.P. unit – Laboratory of Microbiology, University of Ghent, Ghent B-9000, Belgium;

[4]Laboratory of Functional and Evolutionary Morphology, University of Liège, Liège B-4000, Belgium;

[5]Microbial Processes and Interactions laboratory, TERRA teaching and research centre, Gembloux Agro-Bio Tech, University of Liège, Gembloux B-5030, Belgium;

[6]Mass Spectrometry Laboratory, MolSys Research Unit, University of Liège, 4000 Liège, Belgium;

[7]Department of Molecular Biotechnology, Institute of Biology, Leiden University, Leiden, The Netherlands;

[8]Departamento de Microbiología y Genética, Universidad de Salamanca, Salamanca, Spain.

[*]Corresponding author: Sébastien Rigali, srigali@uliege.be

Supplementary Material

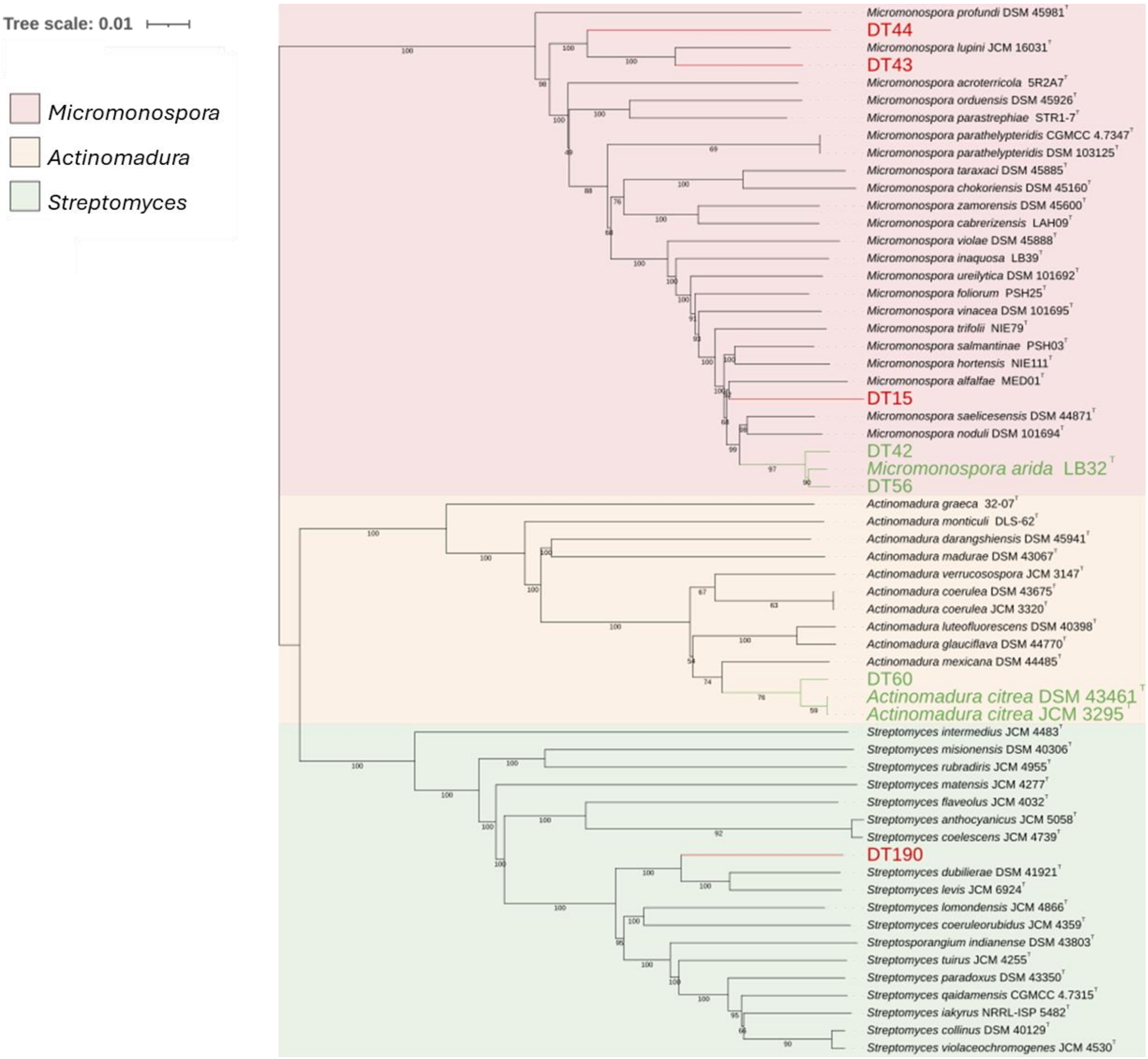

Figure S1. Phylogenomic tree based on whole-genome sequences of isolates DT15, DT42, DT43, DT44, DT56, DT60, and DT190. The tree was inferred using FastME 2.1.6.1 [1] from Genome BLAST Distance Phylogeny (GBDP) distances calculated from genome sequences, with data processed through the Type (Strain) Genome Server (TYGS, [2]) and visualized using the Interactive Tree Of Life (iTOL v6, [3]). Branch lengths are scaled according to the GBDP distance formula d_5. GBDP pseudo-bootstrap support values (numbers below branches) are based on 100 replicates, with an average branch support of 90.6%. The tree was rooted at the midpoint and highlights the species most closely related to the isolates described.

Supplementary Material

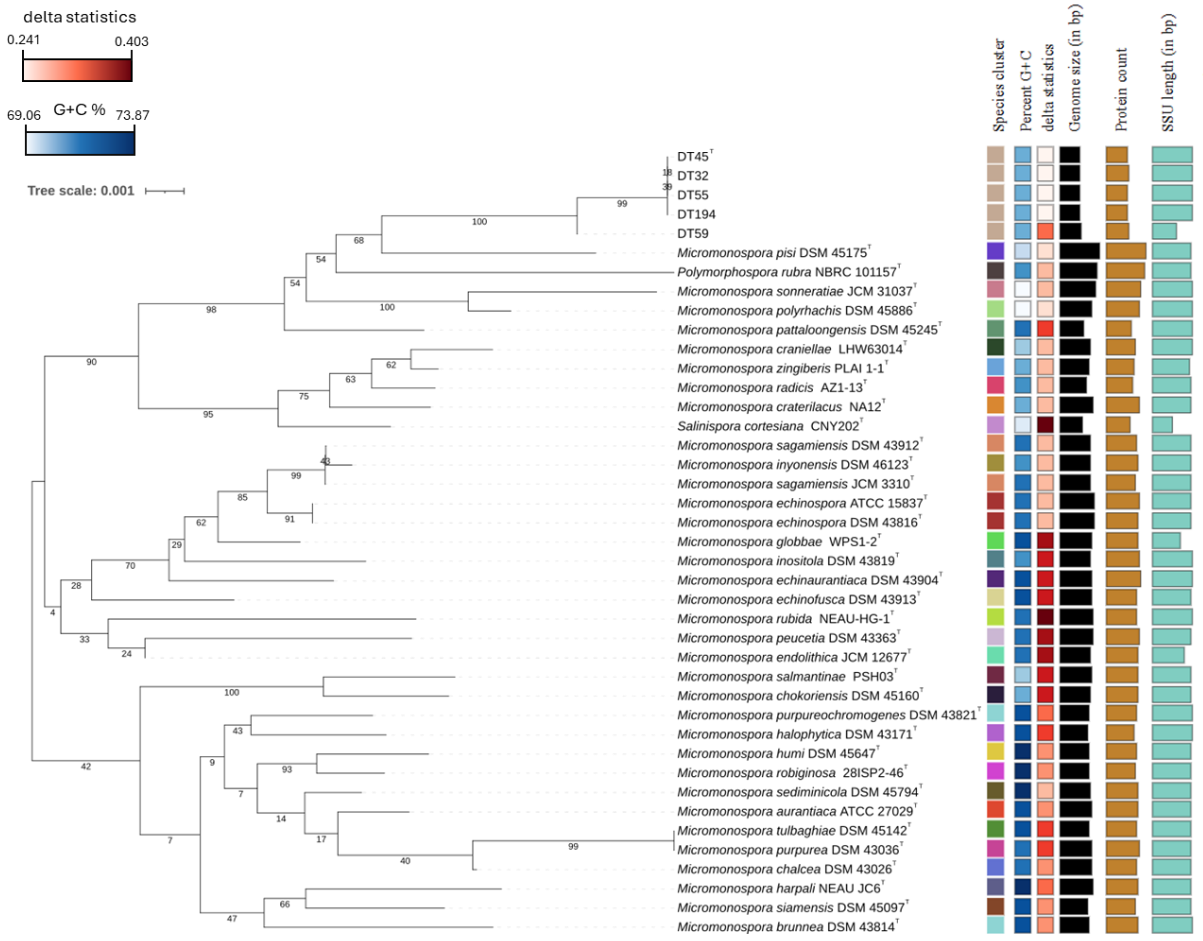

Figure S2. Phylogenomic tree based on 16S rRNA gene sequences of isolate DT45^{T} and related isolates. The tree was inferred using FastME 2.1.6.1 [1] from Genome BLAST Distance Phylogeny (GBDP) distances calculated from 16S rRNA gene sequences, with data processed through the Type (Strain) Genome Server (TYGS, [2]) and visualized using the Interactive Tree Of Life (iTOL v6, [3]). Branch lengths are scaled according to the GBDP distance formula d_5. GBDP pseudo-bootstrap support values (numbers below branches) are based on 100 replicates, with an average branch support of 52.3%. The tree was rooted at the midpoint and highlights the *Micromonospora* spp. most closely related to the described isolates. The genome size is between 4,549,447 bp and 8,739,867 bp. The protein count is between 4,192 and 7,627.

Supplementary Material

Table S1

Genome characteristics

	Genome size (bp)	Genes (total)	Protein-coding genes	G+C content (mol%)	N50	Contigs	CDSs	tRNAs	rRNAs 5S, 16S, 23S	ncRNAs
DT32	4,637,094	4,431	4,302	71.6	881,502	27	4,377	48	1, 1, 1	3
DT45^{T}	4,607,559	4,297	4,177	71.6	700,829	63	4,241	49	1, 1, 2	3
DT55	4,549,447	4,255	4,145	71.7	435,075	36	4,201	48	1, 1, 1	3
DT59	4,691,302	4,460	4,326	71.6	559,659	50	4,405	48	1, 2, 1	3
DT194	4,613,345	4,322	4,194	71.6	668,974	37	4,268	48	1, 1, 1	3

Supplementary Material

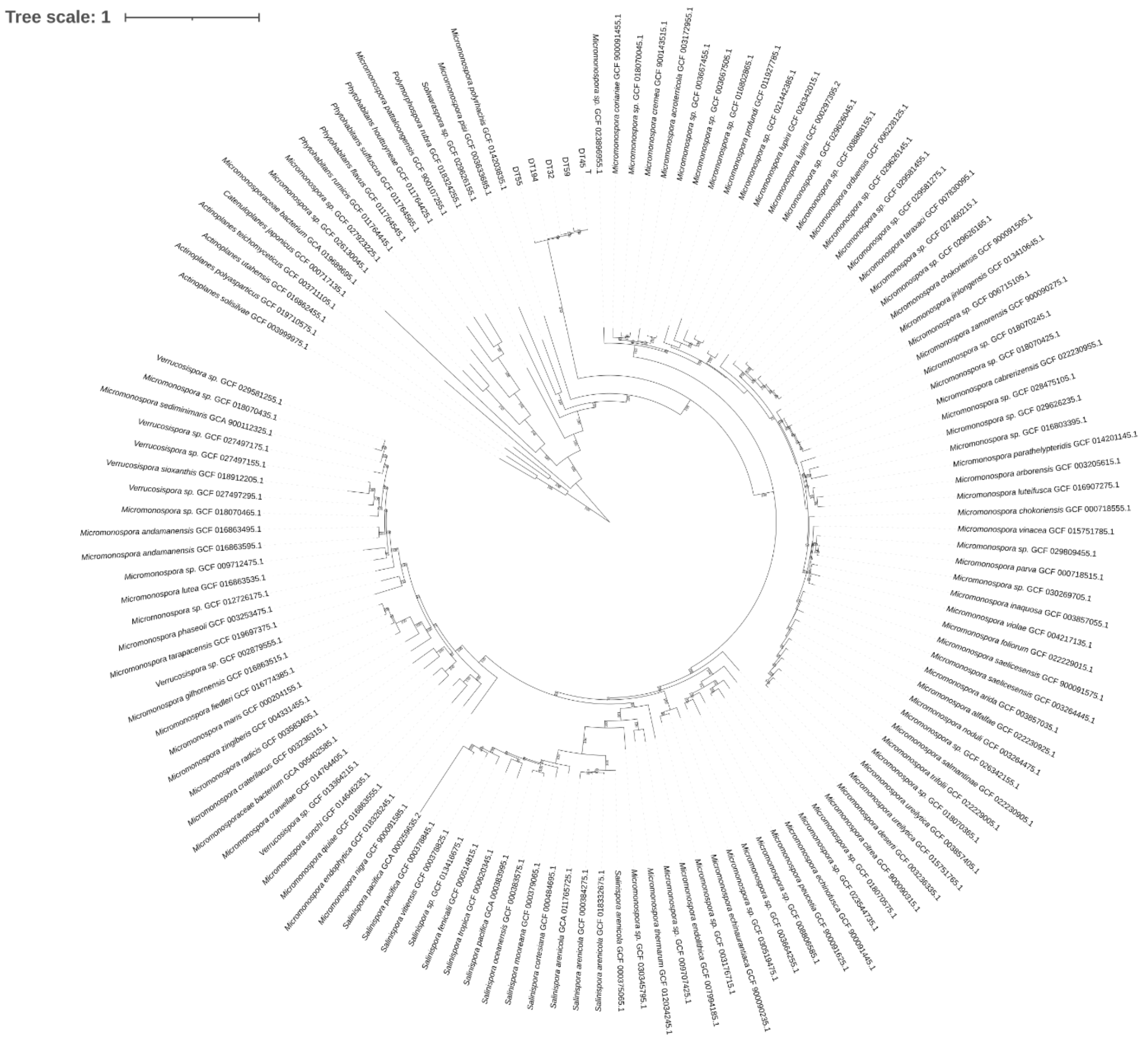

Figure S3. Large phylogenomic tree of isolate DT45^{T} and related isolates. A dataset of 133 genomes was used in the Orthodox and Phylogenomic workflow of GENERA [4] to infer a phylogenomic tree constructed by RaxML [5] using the PROTGAMMALFG model.

Supplementary Material

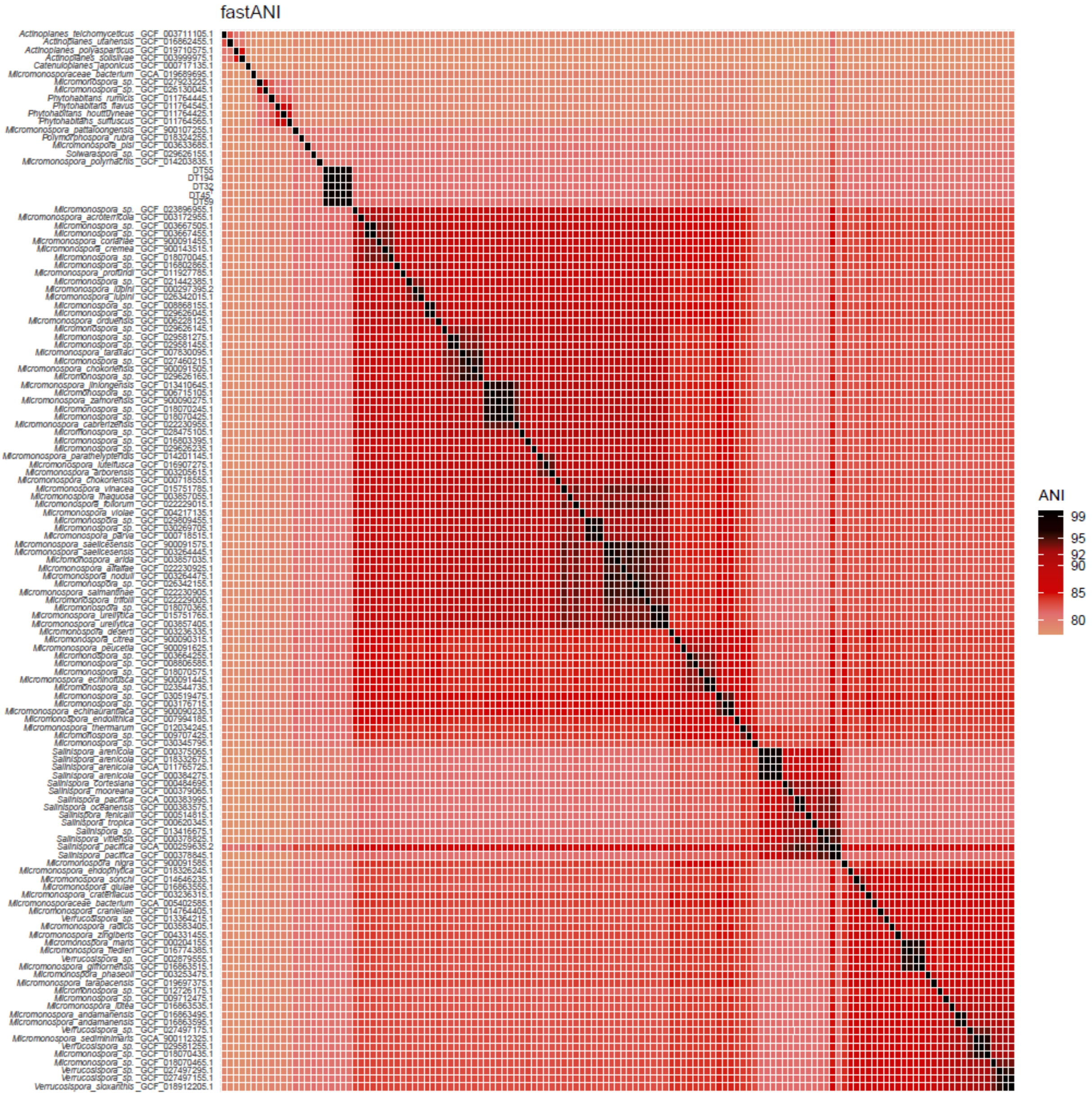

Figure S4. Large Average Nucleotide Identity (ANI) analysis of isolate DT45^{T} and related isolates. A large dataset of 133 genomes was used in the ANI workflow of GENERA [4] to infer a heatmap using FastANI [6] and ggplot2.

Supplementary Material

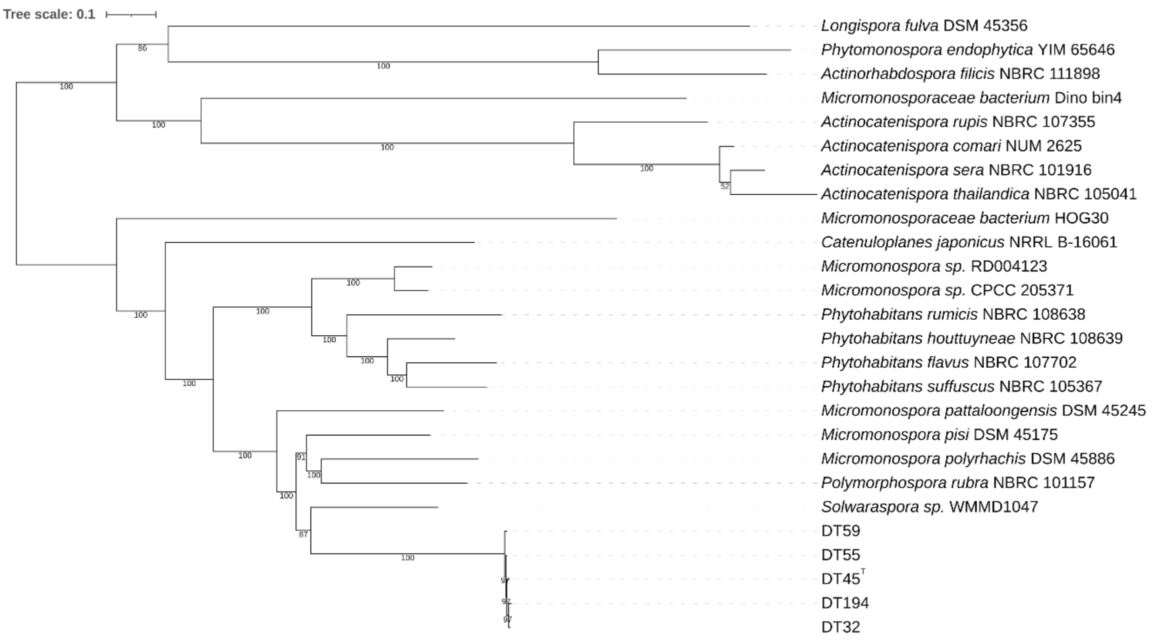

Figure S5. Focused phylogenomic tree of isolate DT45^T and related isolates. A dataset of 26 genomes was used in the Orthodox and Phylogenomic workflow of GENERA [4] to infer a phylogenomic tree constructed by RaxML [5] using the PROTGAMMALFG model.

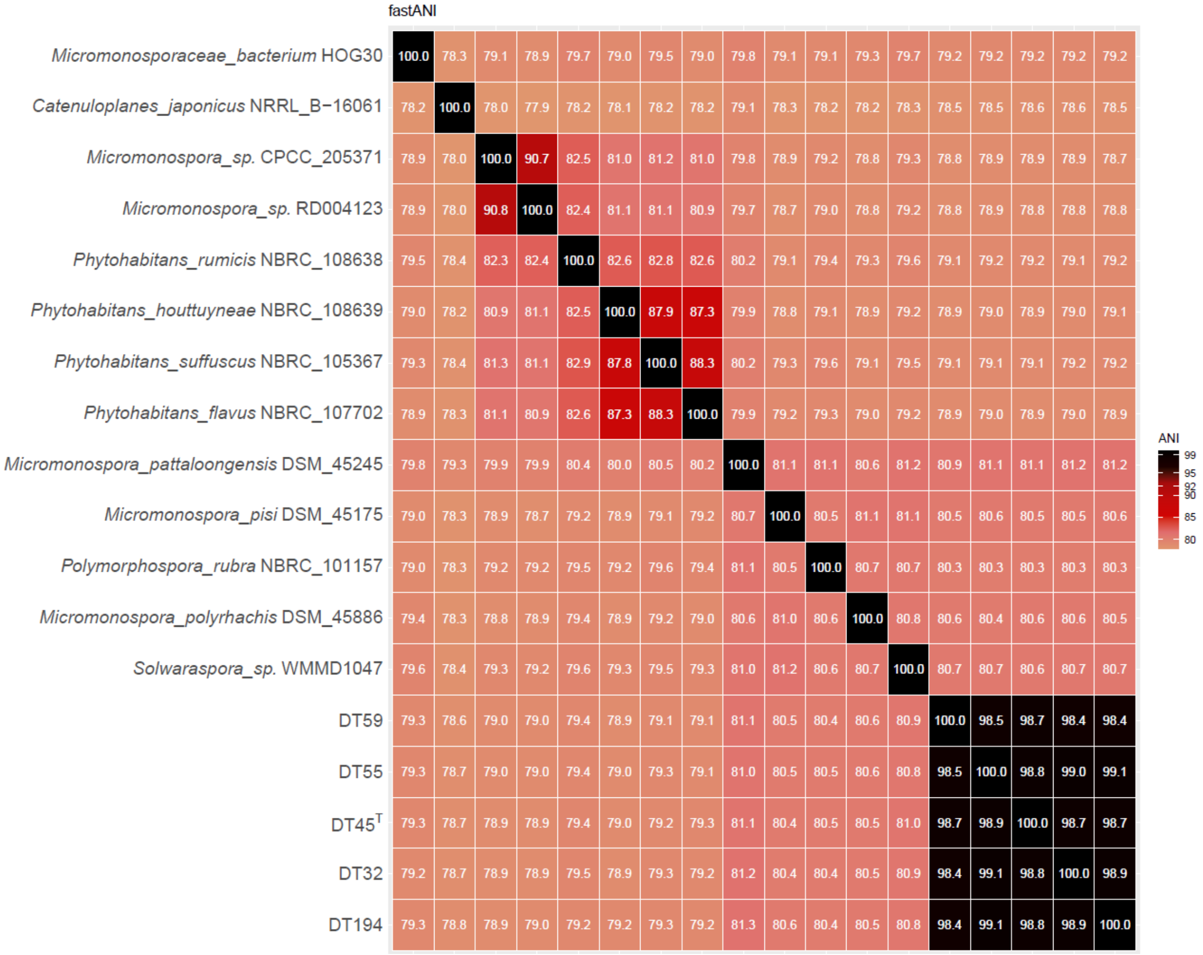

Figure S6. Focused Average Nucleotide Identity (ANI) analysis of isolate DT45^T and related isolates. A focused dataset of 18 genomes was used in the ANI workflow of GENERA [4] to infer a heatmap using FastANI [6] and ggplot2.

Supplementary Material

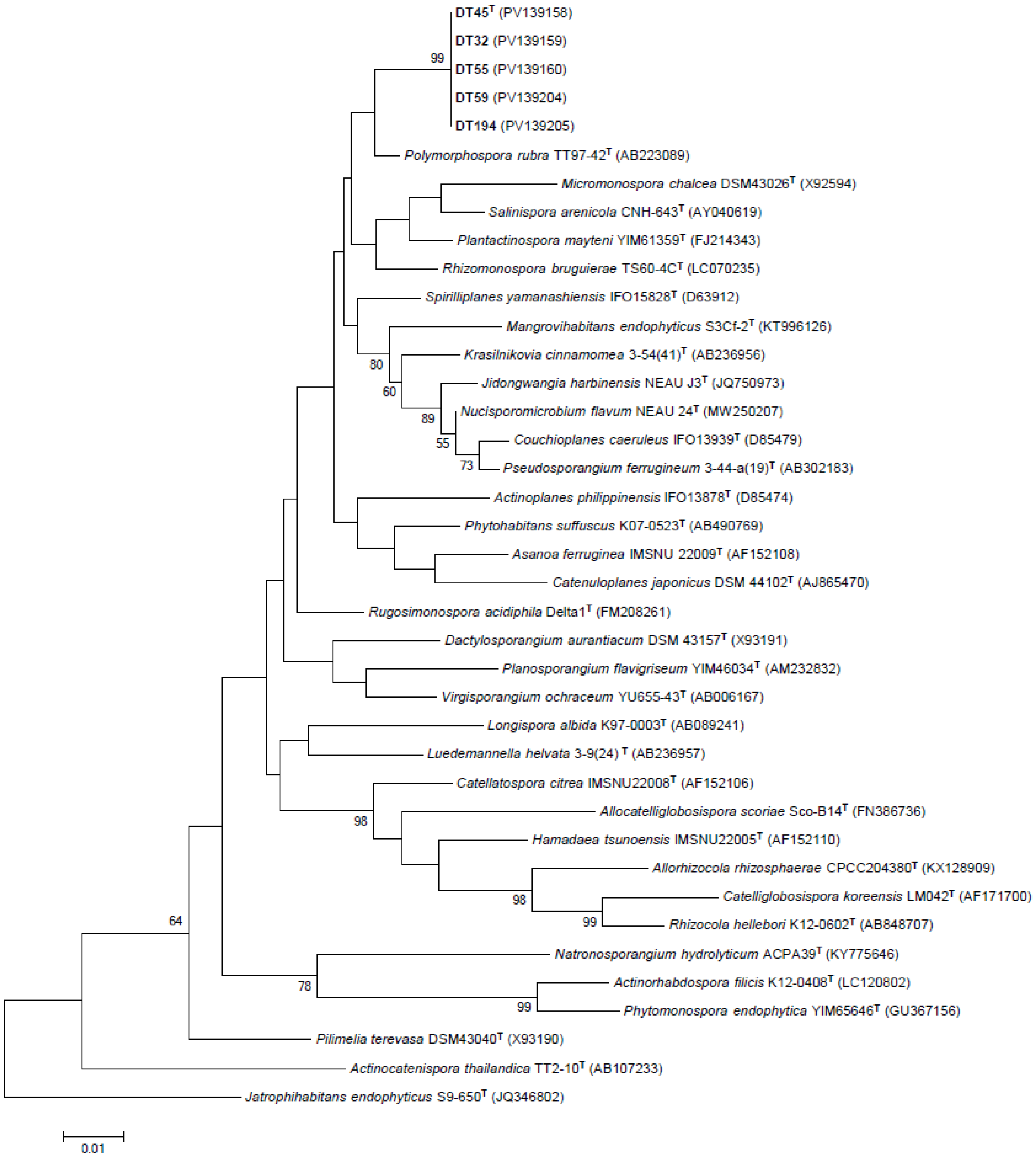

Figure S7. Molecular Phylogenetic analysis by Maximum Likelihood method. The evolutionary history was inferred by using the Maximum Likelihood method based on the Jukes-Cantor model [7]. The tree with the highest log likelihood (-8355.3998) is shown. The percentage of trees in which the associated taxa clustered together is shown next to the branches. Initial tree(s) for the heuristic search were obtained automatically by applying Neighbor-Join and BioNJ algorithms to a matrix of pairwise distances estimated using the Maximum Composite Likelihood (MCL) approach and then selecting the topology with superior log likelihood value. The tree is drawn to scale, with branch lengths measured in the number of substitutions per site. The analysis involved 39 nucleotide sequences. All positions containing gaps and missing data were eliminated. There were a total of 1350 positions in the final dataset. Evolutionary analyses were conducted in MEGA6 [8].

Supplementary Material

Table S2.
Detection of strain DT45^T in metagenome using Protologger

Metagenome	Detection (%)	Mean relative abundance (%)	Standard deviation
wastewater	9	0.19	0.43
activated sludge	3.3	0.02	0.04
human lung	0.2	0.01	0.00
chicken gut	0.8	0.01	0.01
bovine gut	2.8	0.04	0.12
human oral	0.1	0.03	0.00
human vaginal	0.4	0.01	0.00
freshwater	11.8	0.02	0.04
rhizosphere	62.8	0.14	0.37
marine sediment	10.0	0.01	0.01
pig gut	4.0	0.01	0.04
soil	42.5	0.24	0.46
mouse gut	0.3	0.01	0.01
coral	0.8	0.03	0.05
human skin	2.8	0.11	0.25
plant	24.8	0.21	0.33
human gut	0.3	0.01	0.00
marine	1.1	0.03	0.06
insect gut	1.4	0.01	0.01

% of detection of 1,000 amplicon samples.

Supplementary Material

DPG = Diphosphatidylglycerol

PE = Phosphatidylethanolamine

PI = Phosphatidylinositol

GPL = Glycophospholipid

GL = Glycolipid

PL = Phospholipid

L = Lipid

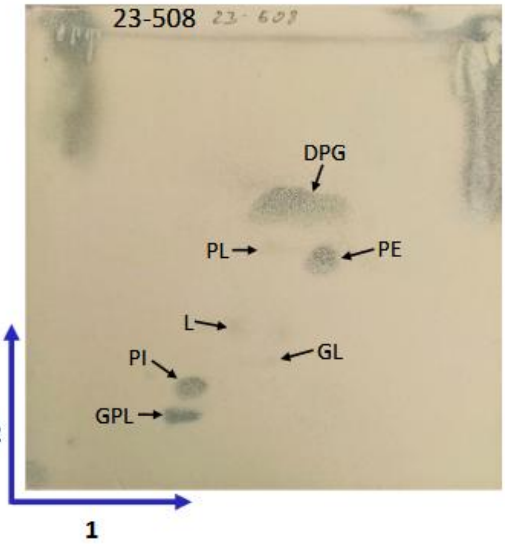

Figure S8. Two-dimensional TLC plate of polar lipids extracted from the strain DT45^{T}.

Supplementary Material

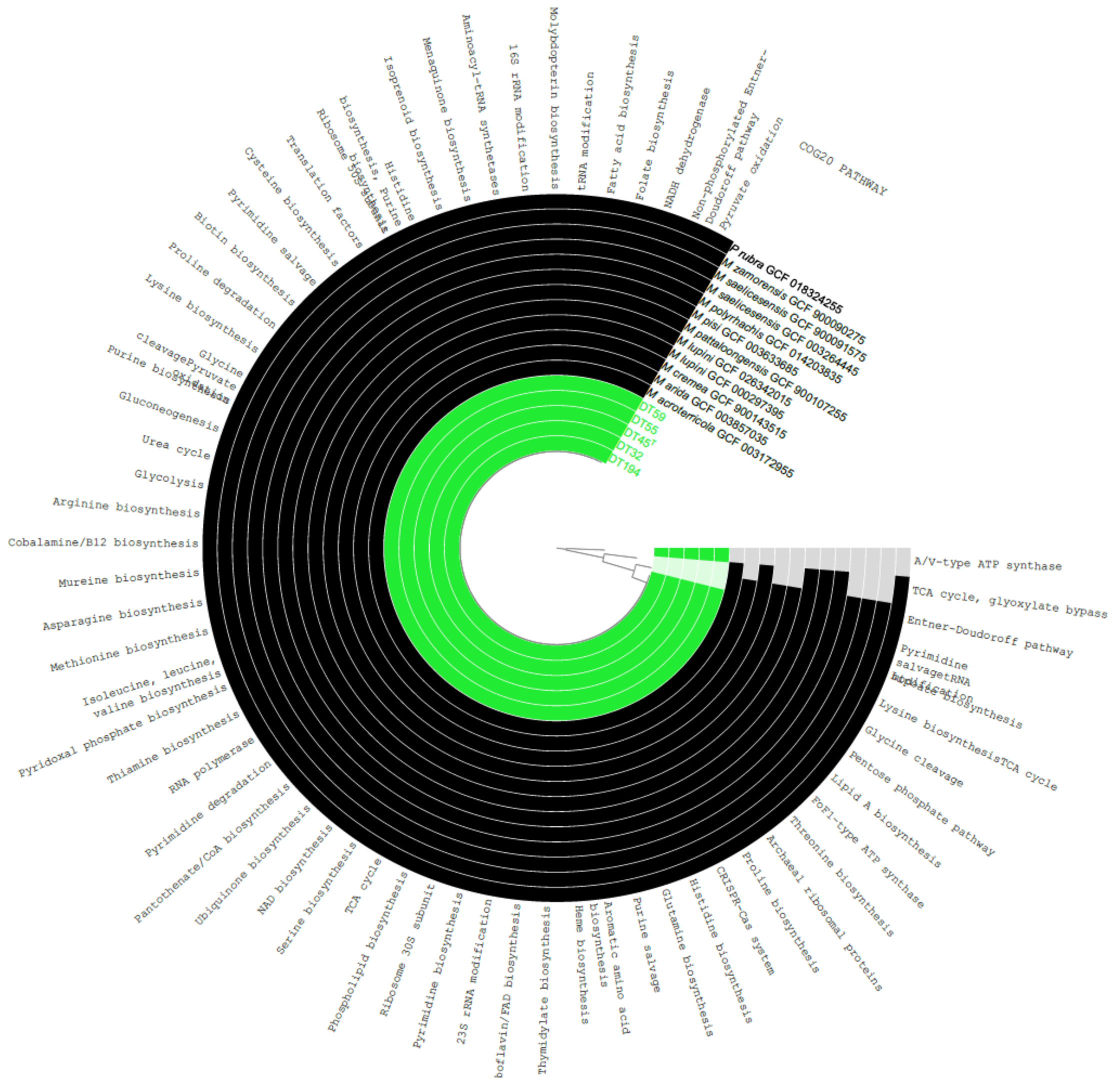

Figure S9. Pangenomics of DT45^T and closest *Micromonospora* strains. The high-resolution figure generated using Anvi'O v8 [9].

Supplementary Material

References

1. **Lefort V, Desper R, Gascuel O**. FastME 2.0: A Comprehensive, Accurate, and Fast Distance-Based Phylogeny Inference Program. *Molecular Biology and Evolution* 2015;32:2798–2800.

2. **Meier-Kolthoff JP, Göker M**. TYGS is an automated high-throughput platform for state-of-the-art genome-based taxonomy. *Nat Commun* 2019;10:2182.

3. **Letunic I, Bork P**. Interactive Tree Of Life (iTOL) v4: recent updates and new developments. *Nucleic Acids Res* 2019;47:W256–W259.

4. **Cornet L, Durieu B, Baert F, D'hooge E, Colignon D, *et al.*** The GEN-ERA toolbox: unified and reproducible workflows for research in microbial genomics. *GigaScience* 2023;12:giad022.

5. **Stamatakis A**. RAxML version 8: a tool for phylogenetic analysis and post-analysis of large phylogenies | Bioinformatics | Oxford Academic. https://academic.oup.com/bioinformatics/article/30/9/1312/238053 (2014, accessed 5 February 2025).

6. **Jain C, Rodriguez-R LM, Phillippy AM, Konstantinidis KT, Aluru S**. High throughput ANI analysis of 90K prokaryotic genomes reveals clear species boundaries. *Nat Commun* 2018;9:5114.

7. **Jukes TH, Cantor CR**. CHAPTER 24 - Evolution of Protein Molecules. In: Munro HN (editor). *Mammalian Protein Metabolism*. Academic Press. pp. 21–132.

8. **Tamura K, Stecher G, Peterson D, Filipski A, Kumar S**. MEGA6: Molecular Evolutionary Genetics Analysis version 6.0. *Mol Biol Evol* 2013;30:2725–2729.

9. **Eren AM, Esen ÖC, Quince C, Vineis JH, Morrison HG, *et al.*** Anvi'o: an advanced analysis and visualization platform for 'omics data. *PeerJ* 2015;3:e1319.
